# Angiopoietin-2 induces angiogenesis via exosomes in human hepatocellular carcinoma

**DOI:** 10.1186/s12964-020-00535-8

**Published:** 2020-03-17

**Authors:** Ji-yan Xie, Jin-xing Wei, Li-hong Lv, Qing-fang Han, Wei-bang Yang, Guo-lin Li, Pan-xia Wang, Shao-bin Wu, Jin-xin Duan, Wen-feng Zhuo, Pei-qing Liu, Jun Min

**Affiliations:** 1 Guangdong Provincial Key Laboratory of Malignant Tumor Epigenetics and Gene Regulation, Sun Yat-Sen Memorial Hospital, Sun Yat-sen University, Guangzhou, 510120 China; 2 Department of Hepatobiliary Surgery, Sun Yat-sen Memorial Hospital, Sun Yat-sen University, Guangzhou, 510120 China; 3 Clinical Trial Institution of Pharmaceuticals, Sun Yat-sen Memorial Hospital, Sun Yat-sen University, Guangzhou, 510120 China; 4 Department of Hepatobiliary and Pancreatic Surgery, The First Affiliated Hospital of Zhengzhou University, No.1, Jianshedong Road, Zhengzhou, 450052 Henan Province China; 5 Laboratory of Pharmacology and Toxicology, School of Pharmaceutical Sciences, Sun Yat-sen University, Guangzhou, 510006 China

**Keywords:** Angiopoietin-2, Angiogenesis, Exosomes, Hepatocellular carcinoma, Endocytosis, Recycling, CRISPR-Cas systems, Epithelial-mesenchymal transition

## Abstract

**Background:**

Hepatocellular carcinoma (HCC) is the most common primary liver cancer and is a highly vascularized solid tumor. Angiopoietin-2 (ANGPT2) has been described as an attractive target for antiangiogenic therapy. Exosomes are small extracellular vesicles secreted by most cell types and contribute to cell-to-cell communication by delivering functional cargo to recipient cells. The expression of ANGPT2 in tumor-derived exosomes remains unknown.

**Methods:**

We detected the ANGPT2 expression in HCC-derived exosomes by immunoblotting, enzyme-linked immunosorbent assay and immunogold labeling, then observed exosomal ANGPT2 internalization and recycling by confocal laser scanning microscopy, co-immunoprecipitation and immunoblotting. We used two HCC cell lines (Hep3B and MHCC97H) to overexpress ANGPT2 by lentivirus infection or knockdown ANGPT2 by the CRISPR/Cas system, then isolated exosomes to coculture with human umbilical vein endothelial cells (HUVECs) and observed the angiogenesis by Matrigel microtubule formation assay, transwell migration assay, wound healing assay, cell counting kit-8 assay, immunoblotting and in vivo tumorigenesis assay.

**Results:**

We found that HCC-derived exosomes carried ANGPT2 and delivered it into HUVECs by exosome endocytosis, this delivery led to a notable increase in angiogenesis by a Tie2-independent pathway. Concomitantly, we observed that HCC cell-secreted exosomal ANGPT2 was recycled by recipient HUVECs and might be reused. In addition, the CRISPR-Cas systems to knock down ANGPT2 significantly inhibited the angiogenesis induced by HCC cell-secreted exosomal ANGPT2, and obviously suppressed the epithelial-mesenchymal transition activation in HCC.

**Conclusions:**

Taken together, these results reveal a novel pathway of tumor angiogenesis induced by HCC cell-secreted exosomal ANGPT2 that is different from the classic ANGPT2/Tie2 pathway. This way may be a potential therapeutic target for antiangiogenic therapy.

Video Abstract

## Background

Hepatocellular carcinoma (HCC) is the most frequent primary liver cancer, the sixth most common neoplasm, and the third leading cause of cancer-related mortality worldwide [1, 2]. HCC usually develops from diverse chronic liver diseases underlying cirrhosis (including chronic viral hepatitis types B and C, alcohol abuse, aflatoxin exposure and nonalcoholic fatty liver disease) [3] and is one of the highly vascularized solid tumors characterized by a high presence of hypervascularity and vascular abnormalities [4]. Antiangiogenic therapy is a vital therapeutic strategy for HCC, especially advanced HCC, and antiangiogenic therapy for the treatment of HCC is well established and accepted [5, 6]. However, the initial resistance or development of resistance remains a major problem, and the reason remains unclear [7, 8]. Additional studies to investigate the mechanism of tumor angiogenesis are needed to better improve the antiangiogenic therapy of HCC.

Angiopoietins are a family of secreted factors comprising angiopoietin-1, angiopoietin-2 (ANGPT2), angiopoietin-3 and angiopoietin-4 (in humans) [9–11]. ANGPT2 has been described as a context-dependent antagonist interfering with angiopoietin-1-induced Tie2 phosphorylation to destroy vascular stability and promote angiogenesis, might confer resistance to antiangiogenic therapy [12], and has emerged as an attractive vascular drug target by blocking the ANGPT2/Tie2 pathway [8, 13]. ANGPT2 has been found to be highly expressed in diverse tumor cells and plays an important role in tumor angiogenesis and inflammation [14, 15]. Studies have revealed that ANGPT2 is highly expressed in HCC and that the level of ANGPT2 is closely related to the development and prognosis of HCC [16, 17].

Exosomes are 30–150 nm extracellular membrane vesicles secreted by most cell types in vivo and in vitro and contribute to cell-to-cell communication by delivering functional proteins, nucleic acids and lipids to recipient cells [18, 19]. Tumor-derived exosomes can be released into the tumor microenvironment (TME) to exert their effects on different cell types (including tumor cells and other non-tumor cells) and influence vascular function, both locally in tumors and remotely in distant organs through the systemic circulation; these exosomes are related to therapy resistance [20, 21]. Accumulating evidence has indicated that HCC-derived exosomes play crucial roles in remodeling TME and promoting tumor angiogenesis [22, 23].

In this study, we found that ANGPT2 existed on HCC-derived exosomes and was delivered into human umbilical vein endothelial cells (HUVECs) via exosome endocytosis to stimulate angiogenesis by a Tie2-independent pathway. Moreover, exosomal ANGPT2 was recycled by recipient HUVECs and might be reused. Conversely, knockdown of exosomal ANGPT2 by the CRISPR/Cas system resulted in an obvious downregulation of angiogenesis induced by HCC-derived exosomes. These results suggest that HCC-derived exosomal ANGPT2 induces tumor angiogenesis by a novel way that is different from the classic ANGPT2/Tie2 pathway of free ANGPT2 to promote tumor progression in HCC.

## Methods

### Cell lines and culture

Hep3B, SNU182, SNU387 and Li7 cells were kindly provided by Stem Cell Bank, Chinese Academy of Sciences (China). MHCC97H and HUVEC cells were purchased from Guangzhou Cellcook Biotech Co., Ltd. (China). Five HCC cell lines, Hep3B, SNU182, SNU387, Li7 and MHCC97H, were cultured in Roswell Park Memorial Institute 1640 medium (RPMI 1640, Gibco, USA) supplemented with 10% fetal bovine serum (FBS, Gibco, USA). HUVECs were cultured in endothelial cell medium (ECM, ScienCell, USA). All cells were maintained in a humidified incubator at 37 °C with 5% CO_2_. The FBS used for exosome isolation was depleted of exosomes by ultracentrifugation for 12 h at 120,000 x g at 4 °C (Optima L-100XP, Beckman, USA). The following sublines were established by infecting cells with the lentiviruses pLV-hANGPT2-mCherry, pLV-mCherry, lentiCRISPRv2-ANGPT2gRNA or lentiCRISPRv2: Hep3B and MHCC97H sublines that stably overexpressed ANGPT2 (named Hep3B-ANGPT2 and MHCC97H-ANGPT2, respectively) and their matched control lines (named Hep3B-CT and MHCC97H-CT, respectively), and those that stably knocked down ANGPT2 (named Hep3B-ANGPT2crispr and MHCC97H-ANGPT2crispr, respectively) and their matched control lines (named Hep3B-V2 and MHCC97H-V2, respectively).

### Statistical analysis

Data are expressed as the mean ± SEM. Statistical analysis of clinical samples was performed by using Welch’s t-tests, and other statistical analyses were performed by using unpaired Student’s t-tests for two groups or ordinary one-way ANOVA with Tukey’s multiple comparison tests for multiple groups (GraphPad Prism 7.0 software, CA, USA). Differences were considered statistically significant at *P* < 0.05 (*P < 0.05, **P < 0.01, ***P < 0.001).

Details regarding vectors and inhibitors, exosome Isolation and characterization, immunoblotting, co-immunoprecipitation (co-IP), transmission electron microscopy (TEM), immunohistochemistry (IHC), enzyme-linked immunosorbent assay (ELISA), immunofluorescence and confocal laser scanning microscopy, Matrigel microtubule formation assay, transwell migration assay, cell counting kit-8 assay (CCK-8), wound healing assay, and the in vivo tumorigenesis assay are provided in Additional file 1: Supporting Materials and Methods.

## Results

### ANGPT2 exists on HCC-derived exosomes

**Fig. 1:**
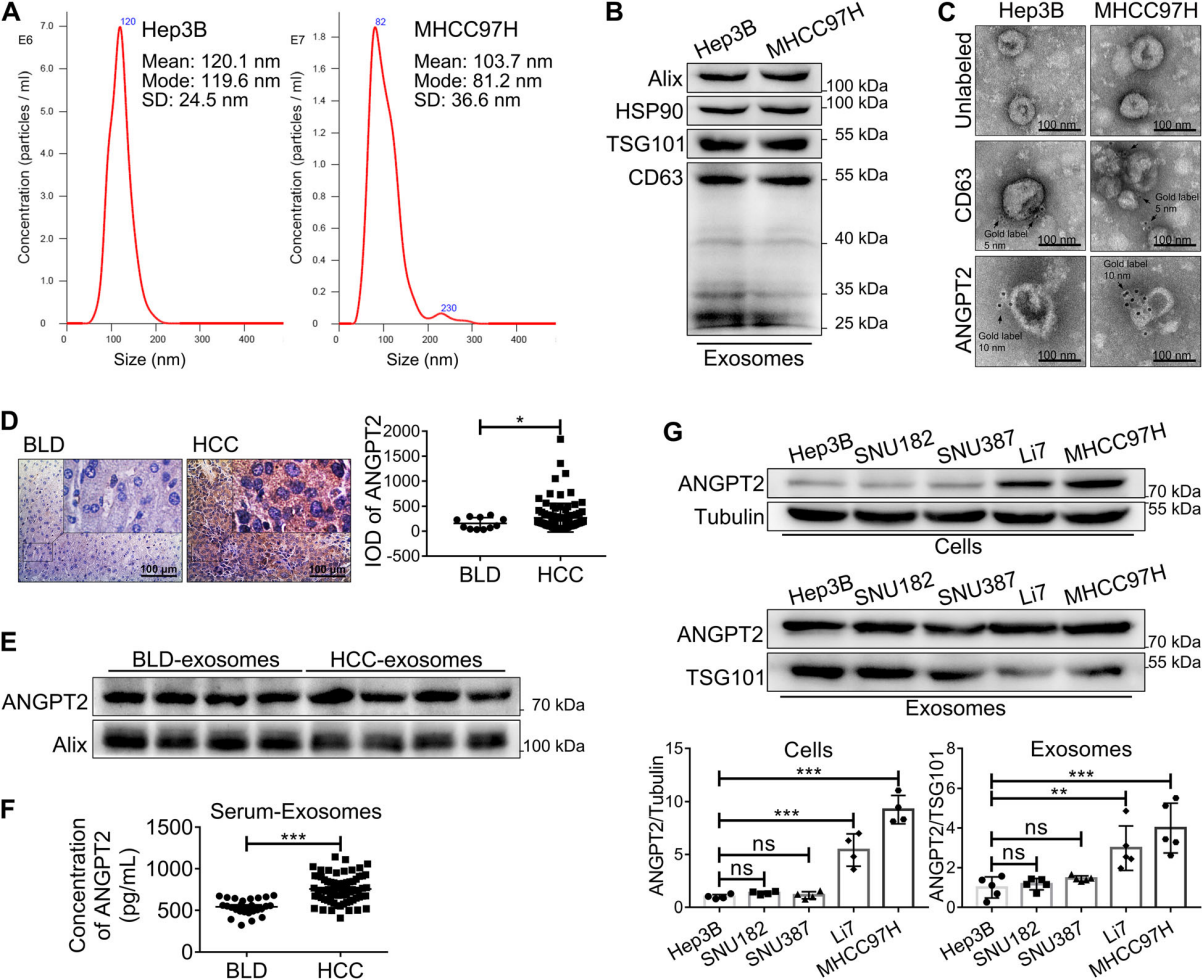
ANGPT2 exists on HCC-derived exosomes. **a** NTA displayed that the majority of isolated exosomes were within 30–150 nm, which is the typical size of exosomes. **b** Immunoblotting showed the typical exosomal markers (Alix, HSP90, TSG101 and CD63) in isolated exosomes. **c** Transmission electron microscopic view of isolated exosomes. The isolated exosomes had cup-shaped morphology, were labeled with exosomal marker CD63 (immunogold = 5 nm), and ANGPT2 was also labeled on isolated exosomes by immunogold (immunogold = 10 nm). Scale bar = 100 nm. **d** IHC demonstrated that the expression of ANGPT2 in HCC tissues (IOD = 270.6 ± 29.36, *n* = 96) was higher than that in BLD tissues (IOD = 157.3 ± 34.9, *n* = 11). Scale bar = 100 μm. *P < 0.05, Welch’s t-tests. **e** Immunoblotting showed that ANGPT2 was positive in exosomes isolated from the sera of both HCC and BLD patients. **f** ELISA showed that the level of exosomal ANGPT2 isolated from the sera of HCC patients (756.5 ± 20.3 pg/mL, *n* = 67) was significantly higher than that from the sera of BLD patients (541.3 ± 18.82 pg/mL, *n* = 26). ***P < 0.001, Welch’s t-tests. **g** Immunoblotting showed the levels of ANGPT2 in different HCC cell lines and their exosomes (Hep3B, SNU182, SNU387, Li7 and MHCC97H), and the levels of HCC cell-secreted exosomal ANGPT2 were consistent with their corresponding cells. *n* = 4 for cell groups, *n* = 5 for exosome groups, **P < 0.01, ***P < 0.001, one-way ANOVA with Tukey’s multiple comparison tests

We identified exosomes isolated from HCC cells by nanoparticle tracking analysis (NTA), immunoblotting and TEM. NTA revealed that most of the exosomes were within 30–150 nm (Fig. 1a), which is the typical size of exosomes. Immunoblotting revealed that isolated exosomes expressed typical exosomal markers, such as Alix, HSP90, TSG101 and CD63 (Fig. 1b). We observed that isolated exosomes had a typical cup-shaped morphology and were labeled with CD63 by TEM (Fig. 1c). Moreover, immunogold labeling showed that ANGPT2 existed on the surface of isolated exosomes without permeabilization by Triton X-100 (Fig. 1c). In addition, we analyzed the expression of ANGPT2 in tissues and serum-exosomes through IHC, immunoblotting and ELISA. The results showed that the expression of ANGPT2 in HCC tissues (IOD = 270.6 ± 29.36, *n* = 96) was higher than that in benign liver disease (BLD) tissues (IOD = 157.3 ± 34.9, *n* = 11) (Fig. 1d); ANGPT2 existed in the serum-exosomes of both HCC and BLD, and exosomal ANGPT2 isolated from serum in HCC (756.5 ± 20.3 pg/mL, *n* = 67) was significantly higher than that in BLD (541.3 ± 18.82 pg/mL, *n* = 26) (Fig. 1e, f). In the in vitro experiment, we detected ANGPT2 levels in five HCC cell lines (Hep3B, SNU182, SNU387, Li7 and MHCC97H) and their exosomes. Immunoblotting showed that ANGPT2 was also carried by exosomes derived from HCC cells, and the levels of exosomal ANGPT2 were consistent with corresponding HCC cells (Fig. 1g). Taken together, these results revealed that ANGPT2 existed on HCC-derived exosomes and had a high level in HCC serum-exosomes.

### HCC cell-secreted exosomal ANGPT2 is delivered into HUVECs via exosome endocytosis

**Fig. 2:**
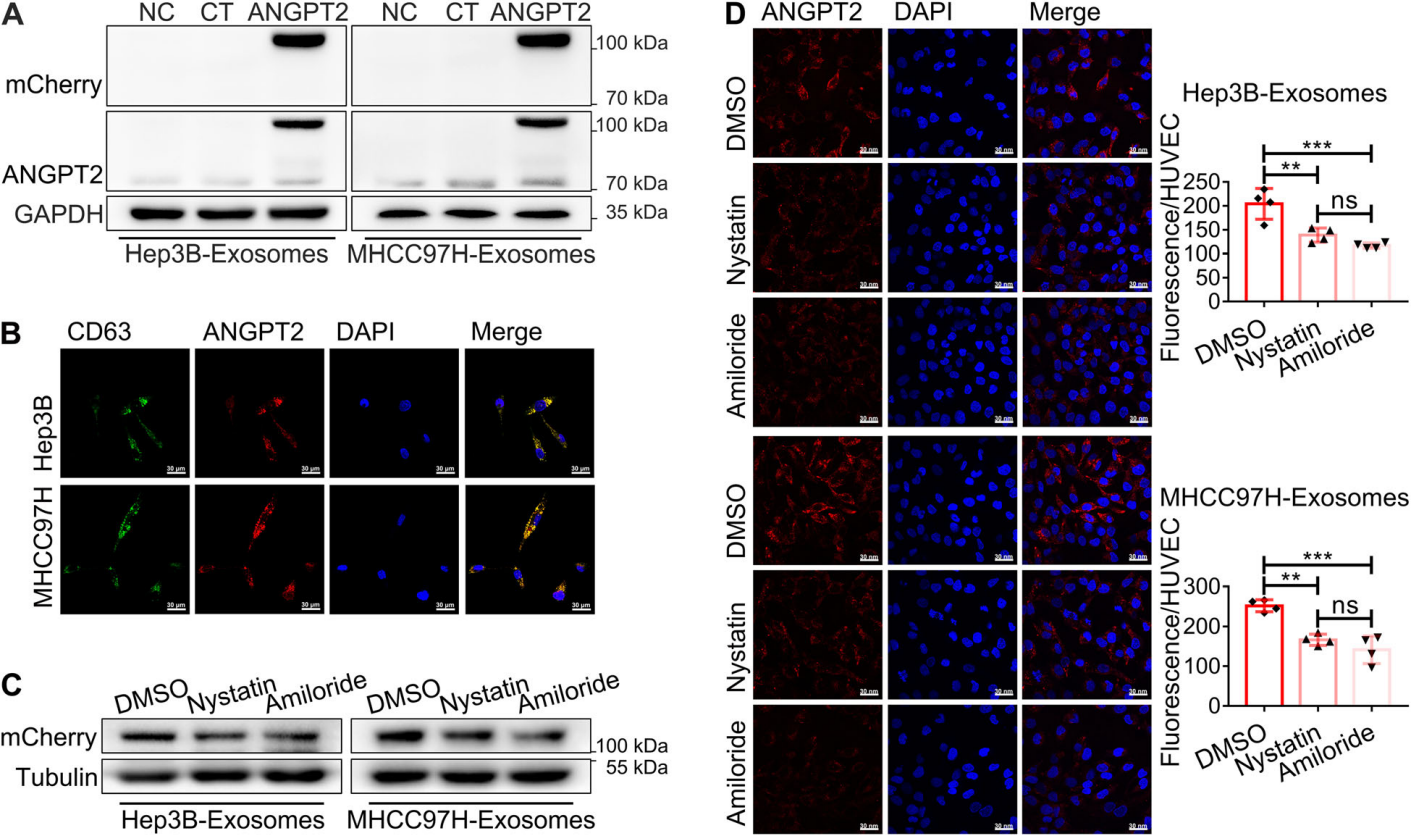
HCC cell-secreted exosomal ANGPT2 is delivered into HUVECs via exosome endocytosis. **a** HUVECs were cultured with or without exosomes derived from ANGPT2-mCherry-expressing HCC cells and their matched control cells for 6 h. Immunoblotting showed that the ANGPT2-mCherry fusion protein was detected in HUVECs. **b** ANGPT2-mCherry-expressing HCC cells were transfected with the pLV-EGFP-CD63 plasmid for 48–72 h to obtain cells that coexpressed the ANGPT2-mCherry and CD63-EGFP fusion proteins. Then, exosomes were isolated from the above HCC cells to be cocultured with HUVECs for 6 h. The confocal laser scanning microscopy observed that ANGPT2-mCherry and CD63-EGFP coexisted and mostly colocalized in HUVECs. Scale bar = 30 μm. **c**, **d** HUVECs were treated with the endocytosis inhibitors nystatin (25 μmol/L), amiloride (100 μmol/L) or control DMSO for 30 min and then cultured with exosomes derived from ANGPT2-mCherry-expressing HCC cells for 6 h. Immunoblotting (**c**) and confocal laser scanning (**d**) showed that the ANGPT2-mCherry level in HUVECs treated with nystatin or amiloride was significantly lower than that in the DMSO control group. Scale bar = 30 nm. n = 4 for each group, **P < 0.01, ***P < 0.001, one-way ANOVA with Tukey’s multiple comparison tests

We constructed stable HCC cell lines that expressed the ANGPT2-mCherry fusion protein and found that the ANGPT2-mCherry fusion protein existed in their exosomes as well. ANGPT2-mCherry-expressing exosomes were cocultured with HUVECs for 6 h, and immunoblotting showed that ANGPT2-mCherry was delivered into HUVECs (Fig. 2a). Further, ANGPT-mCherry-expressing HCC cells were transfected with a plasmid to coexpress the CD63-EGFP fusion protein and then isolated exosomes to coculture with HUVECs for 6 h. Confocal laser scanning microscopy revealed that ANGPT2 and CD63 were both internalized and colocalized mostly in HUVECs (Fig. 2b). Moreover, the endocytosis inhibitors nystatin (25 μmol/L) and amiloride (100 μmol/L) were used to treat HUVECs before coculture, and the level of exosomal ANGPT2 was found to be notably decreased in recipient HUVECs (Fig. 2c, d), suggesting that the internalization process of exosomal ANGPT2 was blocked by endocytosis inhibitors. These results indicated that exosomal ANGPT2 was delivered into HUVECs from HCC cells via exosome endocytosis.

### HCC cell-secreted exosomal ANGPT2 is recycled by recipient HUVECs

**Fig. 3:**
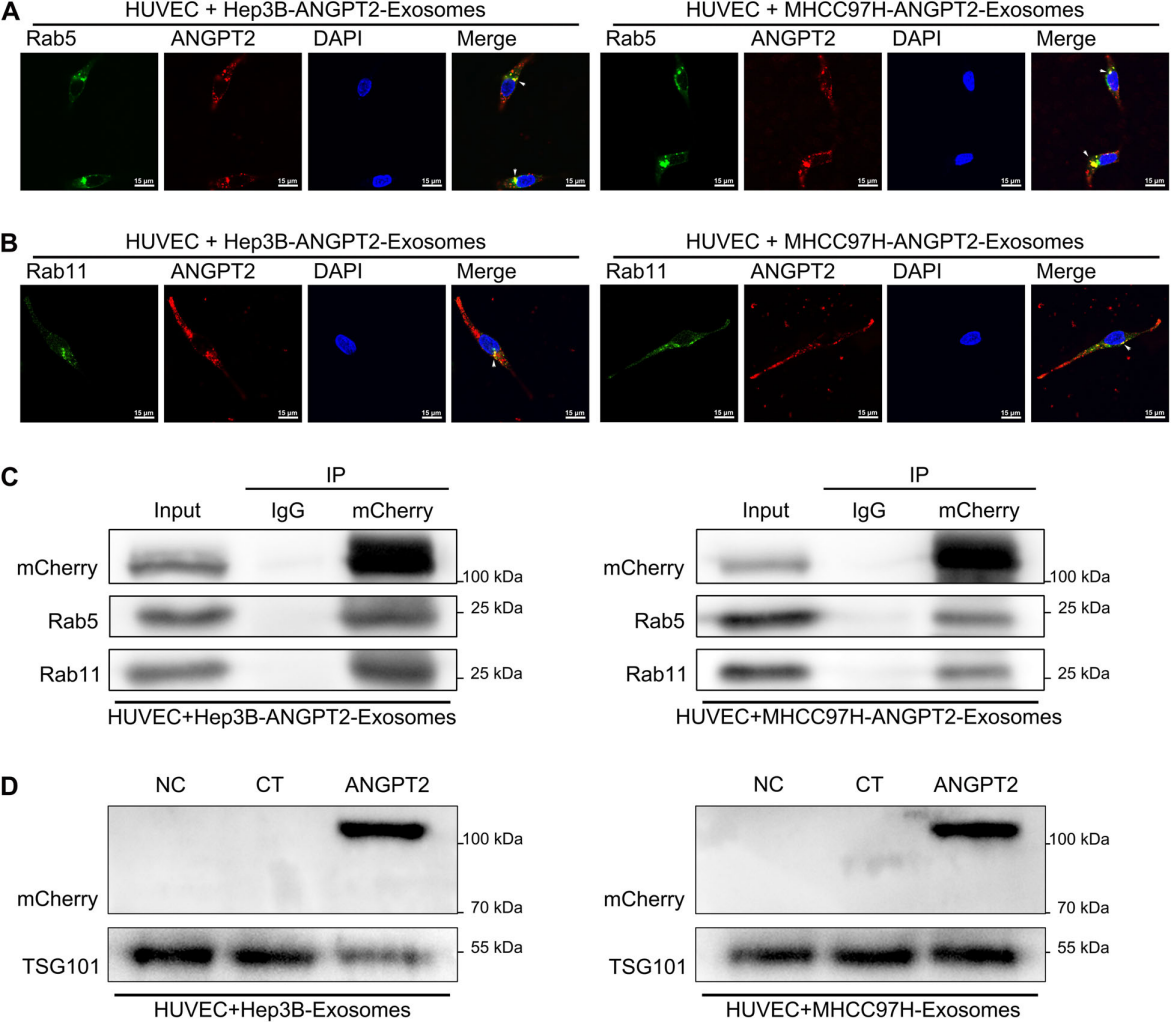
HCC cell-secreted exosomal ANGPT2 is recycled by recipient HUVECs. **a** HUVECs were transfected with the pLV-EGFP-hRab5 plasmid for 48–72 h to express the Rab5-EGFP fusion protein and then cultured with ANGPT2-mCherry-expressing exosomes derived from HCC cells for 11 h. The confocal laser scanning revealed that ANGPT2-mCherry and Rab5-EGFP colocalized near the nucleus in HUVECs. Scale bar = 15 μm. **b** HUVECs were cultured with ANGPT2-mCherry-expressing exosomes derived from HCC cells for 12 h. The confocal laser scanning microscopy observed that ANGPT2-mCherry and Rab11-EGFP also colocalized in HUVECs by immunofluorescent staining using Rab11 antibody. Scale bar = 15 μm. **c** HUVECs were cultured with ANGPT2-mCherry-expressing exosomes derived from HCC cells for 12 h. The co-IP assay showed that exosomal ANGPT2-mCherry had interaction with Rab5 and Rab11 in HUVECs. **d** HUVECs were cultured with or without HCC cell-secreted exosomes for 6 h, then washed with PBS for 3 times and cultured with fresh medium supplemented with 10% exosome-depleted FBS for 12 h. Immunoblotting showed that ANGPT2-mCherry was positive in exosomes derived from HUVECs which had been cultured with ANGPT2-mCherry-expressing exosomes


Interestingly, exosomal ANGPT2 and CD63 separated as time went on, and unlike CD63, exosomal ANGPT2 existed in recipient HUVECs up to 24 h until the end of coculture (Additional file 2: Figure S1). To determine the potential behavior of exosomal ANGPT2 in HUVECs after internalization, HUVECs were transfected with a plasmid to express the Rab5-EGFP fusion protein and then cocultured with ANGPT2-mCherry-expressing exosomes for 11 h. Confocal laser scanning revealed that exosomal ANGPT2 and Rab5 of HUVECs had colocalization near the nucleus in HUVECs (Fig. 3a). The immunofluorescence labeling of Rab11 in HUVECs observed that exosomal ANGPT2 and Rab11 of HUVECs also had colocalization in HUVECs (Fig. 3b). In addition, co-IP displayed that HCC cell-secreted exosomal ANGPT2 had interaction with Rab5 and Rab11 in HUVECs (Fig. 3c). For more direct observation, we used Rab11-EGFP-expressing HUVECs cocultured with ANGPT2-mCherry-expressing exosomes for 12 h, kinetic signal monitoring observed that exosomal ANGPT2, which colocalized with Rab11, was released from live HUVECs (Additional file 3: Video S1; Additional file 4: Video S2; Additional file 5: Figure S2A). To confirm the release of exosomal ANGPT2 from recipient HUVECs, HUVECs were cultured with fresh medium after coculture with ANGPT2-mCherry-expressing exosomes derived from HCC cells for 6 h and the wash with phosphate-buffered saline (PBS), immunoblotting showed that HCC cell-secreted exosomal ANGPT2 was positive in HUVEC-cultured medium and HUVEC-derived exosomes (Additional file 5: Figure S2B; Fig. 3d). These results suggested that HCC cell-secreted exosomal ANGPT2 was recycled by HUVECs after internalization and might be reused.

**Additional file 3: Video S1.** Hep3B cell-secreted exosomal ANGPT2 is recycled by recipient HUVECs. HUVECs were transfected with the pLV-EGFP-hRab11 plasmid for 48–72 h to express the Rab11-EGFP fusion protein and then cultured with ANGPT2-mCherry-expressing exosomes derived from Hep3B cells for 12 h. The kinetic signal monitoring observed that ANGPT2-mCherry, which colocalized with Rab11-EGFP, was released from live HUVECs.



**Additional file 4: Video S2.** MHCC97H cell-secreted exosomal ANGPT2 is recycled by recipient HUVECs. HUVECs were transfected with the pLV-EGFP-hRab11 plasmid for 48–72 h to express the Rab11-EGFP fusion protein and then cultured with ANGPT2-mCherry-expressing exosomes derived from MHCC97H cells for 12 h. The kinetic signal monitoring observed that ANGPT2-mCherry, which colocalized with Rab11-EGFP, was released from live HUVECs.


### The overexpression or knockdown of ANGPT2 in HCC cells and their exosomes

**Fig. 4:**
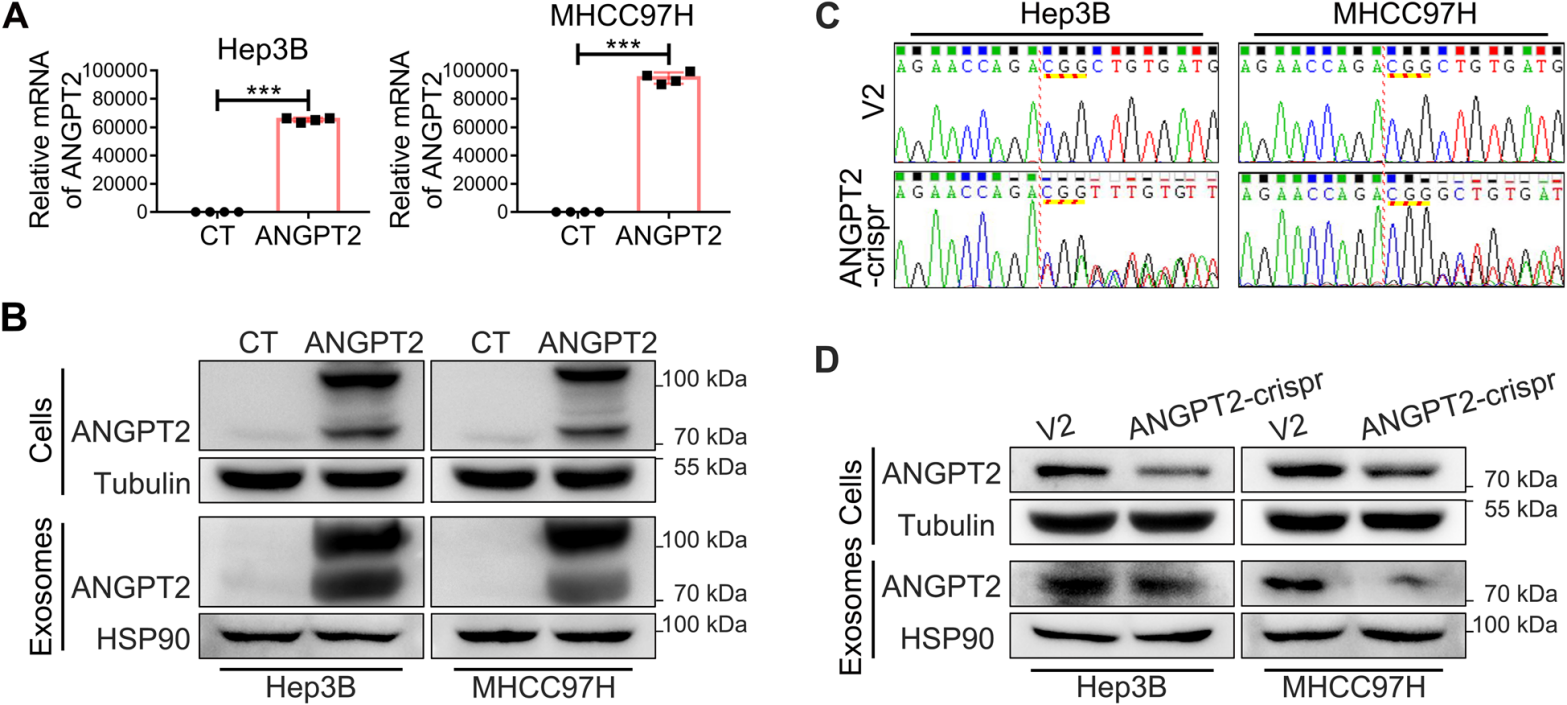
The overexpression or knockdown of ANGPT2 in HCC cells and their exosomes in vitro. **a**, **b** Recombinant lentiviruses that carried the pLV-hANGPT2-mCherry vector or control lentiviruses (only carried pLV-mCherry) were used to infect Hep3B and MHCC97H cells to obtain stable cell lines that overexpressed ANGPT2 fused with mCherry (named Hep3B-ANGPT2 and MHCC97H-ANGPT2, respectively) and matched control stable cell lines (named Hep3B-CT and MHCC97H-CT, respectively). Real-time qPCR detected the relative mRNA levels of ANGPT2 in the stable cell lines (**a**). Immunoblotting showed the expression levels of ANGPT2 in the stable cell lines and their exosomes, which showed that the levels of exosomal ANGPT2 were increased correspondingly (**b**). *n* = 4 for each group, ***P < 0.001, Student’s t-tests. **c**, **d** Recombinant lentiviruses that carried lentiCRISPRv2-ANGPT2gRNA or control lentiviruses (only carried lentiCRISPRv2) were used to infect Hep3B and MHCC97H cells to obtain stable cell lines that knocked down ANGPT2 (named Hep3B-ANGPT2crispr and MHCC97H-ANGPT2crispr, respectively) and matched control stable cell lines (named Hep3B-V2 and MHCC97H-V2, respectively). Genomic DNA sequencing detected mutations in the ANGPT2 gene in stable cell lines (**c**). Immunoblotting showed the expression levels of ANGPT2 in the stable cell lines and their exosomes, which showed that the levels of exosomal ANGPT2 were decreased correspondingly (**d**)

We used two HCC cell lines (Hep3B and MHCC97H) to overexpress ANGPT2 by lentivirus infection or knockdown ANGPT2 by the CRISPR/Cas system. ANGPT2 overexpression efficiency was detected by qPCR (Fig. 4a) and immunoblotting (Fig. 4b), and ANGPT2 deficient efficiency was detected by genomic DNA sequencing (Fig. 4c) and immunoblotting (Fig. 4d), which showed that the levels of exosomal ANGPT2 were increased or decreased correspondingly (Fig. 4b, d). And in the in vivo tumorigenesis assay, the ANGPT2 levels of serum-exosomes had the similar changes (Additional file 6: Figure S3).

### HCC cell-secreted exosomal ANGPT2 promotes the angiogenesis of HUVECs in vitro

**Fig. 5:**
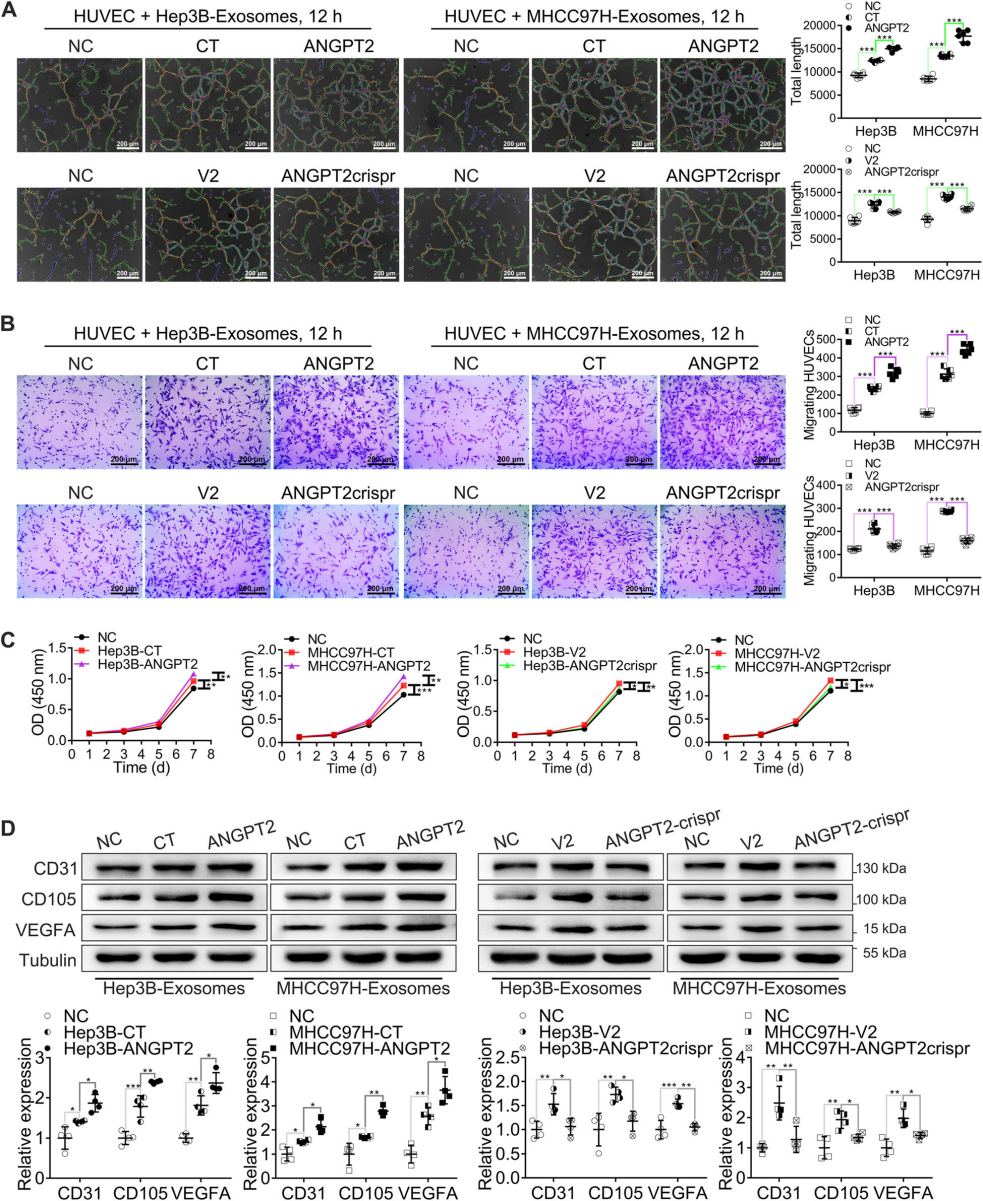
HCC cell-secreted exosomal ANGPT2 promotes the angiogenesis capability of HUVECs in vitro. **a** HUVECs were cultured with or without HCC cell-secreted exosomes for 12 h. The Matrigel microtubule formation assay showed that ANGPT2-overexpressing exosomes significantly promoted tubule formation of HUVECs, and compared with control exosomes, ANGPT2-deficient exosomes abrogated exosome-induced tubule formation. **b** HUVECs were cultured with or without HCC cell-secreted exosomes for 12 h. The transwell migration assay showed that ANGPT2-overexpressing exosomes significantly promoted migration of HUVECs, and compared with control exosomes, ANGPT2-deficient exosomes abrogated exosome-induced migration. **c** HUVECs were cultured with or without HCC cell-secreted exosomes for 7 d and were counted by measuring the OD at 450 nm at 1, 3, 5, and 7 d. CCK-8 showed that HUVEC proliferation was significantly increased after coculture with ANGPT2-overexpressing exosomes, and compared with the coculture with control exosomes, ANGPT2-deficient exosomes abrogated exosome-induced proliferation. **d** HUVECs were cultured with or without HCC cell-secreted exosomes for 72 h. Immunoblotting showed that ANGPT2-overexpressing exosomes notably increased the levels of tumor angiogenesis-related proteins (CD31, CD105 and VEGFA) in HUVECs, and the promotional effect of ANGPT2-deficient exosomes on these angiogenesis-related proteins was notably reduced compared to the promotional effect of control exosomes. Scale bar = 200 μm. *n* = 6 for each group (**a**, **b**), *n* = 4 for each group (**c**, **d**), *P < 0.05, **P < 0.01, ***P < 0.001, one-way ANOVA with Tukey’s multiple comparison tests

We used the Matrigel microtubule formation assay, transwell migration assay, wound healing assay and CCK-8 to observe the angiogenesis capability of HUVECs in vitro. The results showed that tubule formation, migration and proliferation of HUVECs were all significantly increased after coculture with exosomes derived from HCC cells, and the increase induced by MHCC97H-exosomes was obviously higher than that induced by Hep3B-exosomes (Additional file 7: Figure S4). Previous results showed that the level of exosomal ANGPT2 derived from MHCC97H was significantly higher than that from Hep3B (Fig. 1g). However, whether the different effect of MHCC97H-exosomes and Hep3B-exosomes on HUVEC angiogenesis was associated with ANGPT2 was not clear. To determine the role of HCC cell-secreted exosomal ANGPT2 in angiogenesis, we isolated ANGPT2-overexpressing or ANGPT2-deficient exosomes from HCC cells to coculture with HUVECs and observed the angiogenesis capability of HUVECs. The results showed that ANGPT2-overexpressing exosomes notably promoted the tube formation, migration, and proliferation of HUVECs, and ANGPT2-deficient exosomes abrogated exosome-induced promotions of these capabilities (Fig. 5a-c; Additional file 8: Figure S5). In addition, the levels of tumor angiogenesis-related proteins CD31, CD105 and VEGFA in HUVECs were all obviously increased after coculture with ANGPT2-overexpressing exosomes, and compared with control exosomes, ANGPT2-deficient exosomes abrogated exosome-induced increases of these angiogenesis-related proteins (Fig. 5d). These data indicated that HCC cell-secreted exosomes promoted angiogenesis of HUVECs and that exosomal ANGPT2 played a crucial role in this angiogenesis process.

Furthermore, the time-course experiment displayed that the phosphorylation of Tie2 and downstream p85 subunit of phosphatidylinositol 3-kinase (PI3Kp85) had no obvious changes after coculture with ANGPT2-overexpressing exosomes derived from HCC cells for 15 min, 30 min, 1 h, 2 h, 4 h, and 6 h respectively (Additional file 9: Figure S6), suggesting that the angiogenesis induced by HCC cell-secreted exosomal ANGPT2 was independent from Tie2, which has been usually considered as the receptor of ANGPT2. However, the phosphorylation of AKT (Ser473 and Thr308), eNOS (Ser1177) and β-catenin in HUVECs was markedly increased after coculture with ANGPT2-overexpressing exosomes, and compared with control exosomes, the ANGPT2-deficient exosomes abrogated exosome-induced phosphorylation of these factors (Additional file 10: Figure S7), indicating that HCC cell-secreted exosomal ANGPT2 activated the AKT/eNOS and AKT/β-catenin pathways in HUVECs.

### ANGPT2 promotes malignant progression of HCC

**Fig. 6:**
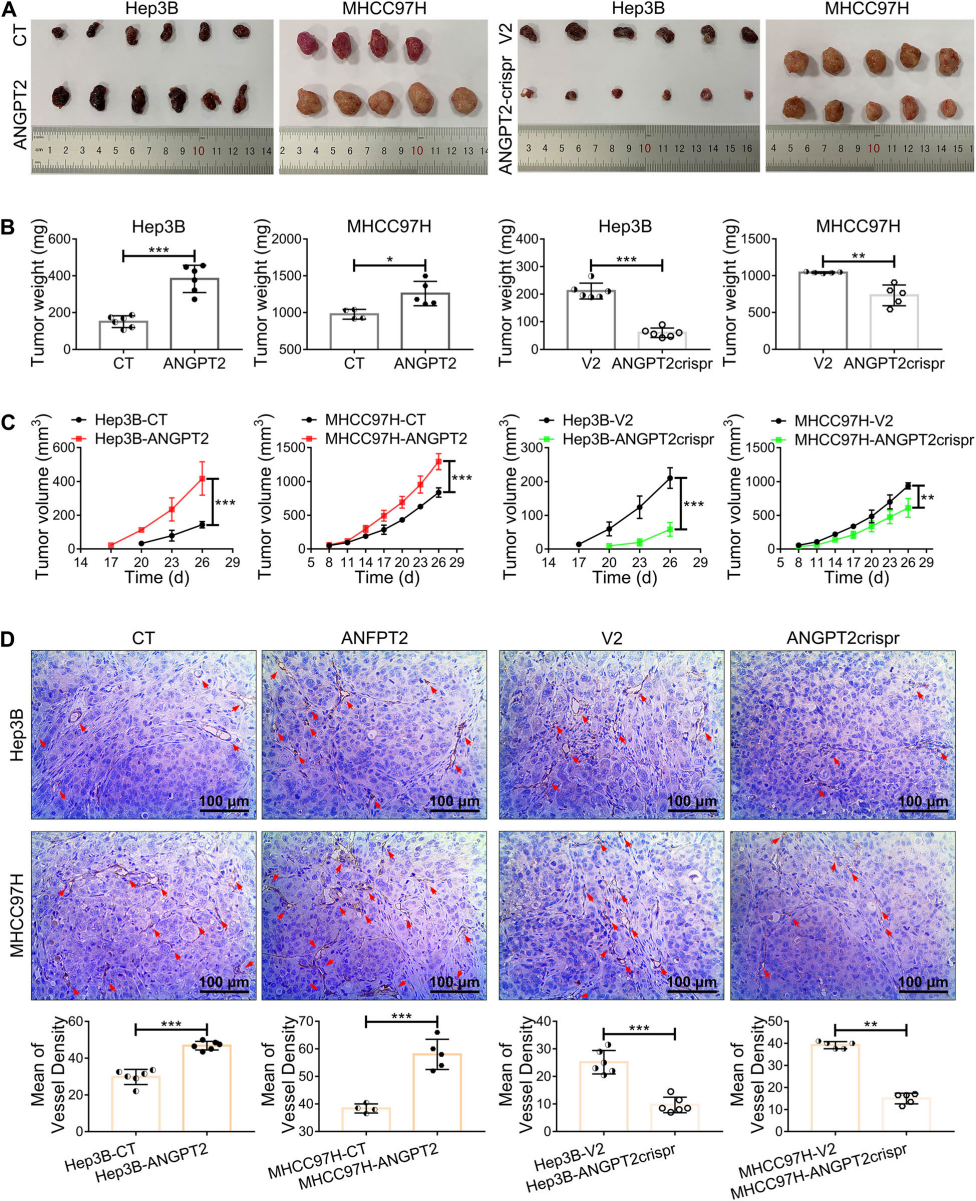
ANGPT2 promotes growth and angiogenesis of HCC in vivo. **a** 1 × 10^7^ ANGPT2-overexpressing or ANGPT2-deficient HCC cells (Hep3B-ANGPT2 and MHCC97H-ANGPT2 or Hep3B-ANGPT2crispr and MHCC97H-ANGPT2crispr, respectively) and their matched control cells (Hep3B-CT, MHCC97H-CT; Hep3B-V2, MHCC97H-V2) were resuspended in 100 μL PBS and were injected subcutaneously into the right flank of 5-week-old male BALB/c nude mice; tumorigenesis was then observed for 26 d. **b** Tumor weight at 26 d after HCC cell implantation. The weight of the ANGPT2-overexpressing group was significantly increased compared with that of the control group, and compared with the control group, the ANGPT2-deficient group had a significant decrease in weight. **c** Tumor volume was measured once every 3 d. The overexpression of ANGPT2 led to a notable increase in tumor volume, and the knockdown of ANGPT2 resulted in an obvious decrease in tumor volume. **d** IHC analysis of the vascular density of tumors was performed by labeling CD31. The overexpression of ANGPT2 led to a notable increase in vascular density, and knockdown of ANGPT2 resulted in a significant decrease in vascular density. *n* = 6 for all Hep3B groups, *n* = 4 for MHCC97H-CT group, *n* = 5 for other MHCC97H groups, *P < 0.05, **P < 0.01, ***P < 0.001, Student’s t-tests

Transwell migration assays, wound healing assays and CCK-8 assays showed that ANGPT2-overexpressing HCC cells had dramatic increases in migration and proliferation compared with those in the control, and these capabilities of ANGPT2-deficient HCC cells were notably reduced (Additional file 11: Figure S8). Moreover, in the in vivo tumorigenesis assay, we found that the overexpression of ANGPT2 led to a significant increase in growth and angiogenesis in HCC, and these capabilities of ANGPT2-deficient HCC were significantly decreased (Fig. 6). And compared with the control group, the ANGPT2-overexpressing group had a high ANGPT2 level of serum-exosomes, the ANGPT2-deficient group had a low ANGPT2 level of serum-exosomes (Additional file 6: Figure S3B). These results suggested that ANGPT2 promoted HCC malignant progression both in vitro and in vivo.

### ANGPT2 increases epithelial-mesenchymal transition (EMT) in HCC

In both the in vitro and in vivo experiments, immunoblotting showed that ANGPT2 overexpression in HCC led to a dramatic increase in the ZEB1, N-cadherin, vimentin, Twist1 and Snail levels and a decrease in the E-cadherin level, suggesting that EMT increased in HCC (Additional file 12: Figure S9A, C). Conversely, EMT of ANGPT2-deficient HCC decreased compared with that of the control (Additional file 12: Figure S9B, C). These data implied that ANGPT2 increased EMT activation in HCC and might be a crucial regulator of EMT.

## Discussion

A study demonstrated that ANGPT2 is stored in and released from endothelial cell Weibel-Palade bodies, the primary endothelial storage granule of the procoagulant von Willebrand factor [24]. However, emerging evidence shows that ANGPT2 can be secreted via exosomes. Currently, ANGPT2 is reported to be a cargo of exosomes derived from endothelial cells, human menstrual blood-derived stem cells, human umbilical cord mesenchymal stem cells, and cardiac mesenchymal stromal cells; in addition, this cargo can be isolated from the serum-exosomes of diabetic patients [25–29]. Many studies have found that ANGPT2 has a high expression level in various tumors [14–17]. However, whether ANGPT2 is carried by tumor-derived exosomes has not been reported. Here, we found that ANGPT2 existed on HCC-derived exosomes (Fig. 1c, e-g). In immunogold labeling, exosomes were not permeabilized, suggesting that ANGPT2 is on the surface of exosomes as a membrane protein (Fig. 1c), which is consistent with the immunoblotting analysis of a previous report [29]. Furthermore, we found that ANGPT2 had a high level in HCC serum-exosomes compared with BLD serum-exosomes (Fig. 1f). Taken together, these results confirm that ANGPT2 exists on the surface of HCC-derived exosomes.

Although a study suggests that exosomal ANGPT2 secretion may be regulated by the PI3K/AKT/eNOS and syndecan-4/syntenin pathways in endothelial cells [29], the behavior of exosomal ANGPT2 after secretion remains unknown. In this study, we found that HCC cell-secreted exosomal ANGPT2 was delivered into HUVECs (Fig. 2a, b), which is completely different from a previous report that ANGPT2 is not internalized into endothelial cells but is instead released from the cell surface into the surrounding medium in the ANGPT2/Tie2 pathway [30]; this result suggests that exosomal ANGPT2 has a novel way to interact with recipient cells that is different from the ANGPT2/Tie2 pathway of free ANGPT2. Moreover, we found that CD63, which is a typical marker of exosomes [19], was also internalized by HUVECs and mostly colocalized with exosomal ANGPT2 in HUVECs after 6 h of coculture (Fig. 2b), suggesting that exosomal ANGPT2 is internalized by HUVECs via exosomes. For exosome internalization, clathrin-mediated endocytosis has been reported to be a way that recipient cells take up exosomes [31]. To determine whether the internalization of exosomal ANGPT2 associates with endocytosis, we used the endocytosis inhibitors nystatin and amiloride to treat HUVECs before coculture [32], and we found that the internalization process of exosomal ANGPT2 was blocked (Fig. 2c, d), suggesting that HCC cell-secreted exosomal ANGPT2 is delivered into HUVECs via exosome endocytosis.

Furthermore, we found that exosomal ANGPT2 remains within recipient cells for an extended period of time (Additional file 2: Figure S1), and there are studies that have also shown that ANGPT2 has a long half-life and may be reused by endothelial cells [24, 30]. To determine whether exosomal ANGPT2 is recycled by recipient cells, we observed the relationship of exosomal ANGPT2 with Rab5 and Rab11, which are crucial participants in the cell recycling process [33]. The results showed that HCC cell-secreted exosomal ANGPT2 had colocalization and interaction with Rab5 and Rab11 in recipient HUVECs (Fig. 3a-c) and was released from HUVECs (Fig. 3d; Additional file 3: Video. S1; Additional file 4: Video S2; Additional file 5: Figure S2), indicating that exosomal ANGPT2 is recycled and may be reused by recipient cells. This result extends our understanding of the pathway by which exosomal ANGPT2 interacts with recipient cells, and suggests that HCC-derived exosomal ANGPT2 may have important effect on the recipient HUVECs.

Accumulating evidence has indicated that HCC-derived exosomes may play important roles in tumor angiogenesis by delivering microRNA, proteins and other cargoes to communicate with cells in TME and remold TME [34–36]. Here, we also found that HCC cell-secreted exosomes promoted the angiogenesis of HUVECs (Additional file 7: Figure S4). Additionally, we found that ANGPT2-overexpressing exosomes dramatically promoted HUVEC angiogenesis, and compared with control exosomes, ANGPT2-deficient exosomes abrogated exosome-induced angiogenesis (Fig. 5; Additional file 8: Figure S5), indicating that HCC cell-secreted exosomal ANGPT2 promotes angiogenesis. Furthermore, we found that the phosphorylation of Tie2 and downstream PI3Kp85 had no obvious changes after coculture with HCC cell-secreted exosomal ANGPT2 (Additional file 9: Figure S6), and that HCC cell-secreted exosomal ANGPT2 activated the AKT/eNOs and AKT/β-catenin pathways in HUVECs (Additional file 10: Figure S7), indicating that HCC cell-secreted exosomal ANGPT2 may induce angiogenesis by activating the AKT/eNOs and AKT/β-catenin pathways instead of interfering the phosphorylation of Tie2 and PI3Kp85.

The CRISPR/Cas system is widespread for genome engineering to activate or repress gene expression and has promising prospects for use in cancer research by providing an efficient technology to dissect mechanisms of tumorigenesis, identify targets for drug development, and arm cells for cell-based therapies [37]. In this study, the CRISPR/Cas system was constructed to knockdown ANGPT2 (Fig. 4c, d), and it has not yet been reported. Here we found that ANGPT2 promoted the malignant progression of HCC (Fig. 6; Additional file 11: Figure S8), and this promotion was consistent with the EMT activation (Additional file 12: Figure S9). This result is accordant with studies on other tumors that ANGPT2 promotes tumor development by increasing EMT, including breast cancer, oral squamous cell carcinoma and lung cancer [38–41]. These studies underscore the contribution of ANGPT2 in tumor progression, not only by stimulating angiogenesis but also by promoting EMT. However, the knockdown of ANGPT2 by the CRISPR/Cas system significantly suppressed the HCC progression, not only by decreasing the level of exosomal ANGPT2 but also by inhibiting EMT activation, suggesting that the CRISPR/Cas system is a promising trend of HCC therapy by blocking ANGPT2.

In the in vivo tumorigenesis assay, Hep3B cells were more responsive to ANGPT2 change than MHCC97H cells, this might be related to the malignancy of HCC cells. Although both Hep3B and MHCC97H were tumorigenic in nude mice, Hep3B had a longer latency period and smaller volume compared with MHCC97H (Fig. 6). In addition, there are studies indicating that Hep3B cell line is low-metastatic and MHCC97H cell line is high-metastatic [42, 43]. However, further explorations are needed to determine whether ANGPT2 as an antiangiogenic therapy is more suitable towards a specific type of HCC.

## Conclusions

Although the treatment of HCC has evolved considerably, novel targets and prognosis predictors are still needed to improve survival. Patients with HCC, especially advanced HCC, benefit from antiangiogenic therapy, but the resistance still remains. ANGPT2 has been found to play a crucial role in angiogenesis and might confer resistance to antiangiogenic therapy. Recent studies targeting ANGPT2 to antiangiogenesis have focused on blocking the ANGPT2/Tie2 pathway. This study indicates that HCC cell-secreted exosomal ANGPT2 has a novel pathway to induce tumor angiogenesis that is different from the classic ANGPT2/Tie2 pathway of free ANGPT2 (Additional file 13: Figure S10); targeting this way may improve current antiangiogenic therapies. Furthermore, the CRISPR/Cas system to block ANGPT2 is a promising therapeutic method in HCC, not only by inhibiting angiogenesis, but also by suppressing EMT activation.

## Supplementary information

**Additional file 1.** Supporting materials and methods.

**Additional file 2: Figure S1.** HCC cell-secreted exosomal ANGPT2 exists in recipient HUVECs for a long time. ANGPT2-mCherry-expressing HCC cells were transfected with pLV-EGFP-CD63 plasmid for 48–72 h to obtain cells that coexpressed the ANGPT2-mCherry and CD63-EGFP fusion proteins. Exosomes isolated from the above HCC cells were cocultured with HUVECs for 6 h and then removed. We observed that ANGPT2-mCherry and CD63-EGFP separated as time went on, and different from exosomal CD63, exosomal ANGPT2 existed in recipient HUVECs up to 24 h by confocal laser scanning microscopy at 8, 12 and 24 h, respectively. Scale bar = 15 μm.

**Additional file 5: Figure S2.** HCC cell-secreted exsomal ANGPT2 is released from recipient HUVECs. **(A)** HUVECs were transfected with the pLV-EGFP-hRab11 plasmid for 48–72 h to express the Rab11-EGFP fusion protein and then cultured with ANGPT2-mCherry-expressing exosomes derived from HCC cells for 12 h. The kinetic signal monitoring observed that ANGPT2-mCherry, which colocalized with Rab11-EGFP, was released from live HUVECs. Scale bar = 15 μm. **(B)** HUVECs were cultured with or without HCC cell-secreted exosomes for 6 h, then washed with PBS for 3 times and cultured with fresh medium supplemented with 10% exosome-depleted FBS for 12 h. Immunoblotting showed that ANGPT2-mCherry was positive in medium cultured with HUVECs which had been cultured with ANGPT2-mCherry-expressing exosomes.

**Additional file 6: Figure S3.** The overexpression or knockdown of ANGPT2 in HCC tissues and serum-exosomes in vivo. The ANGPT2-overexpressing, ANGPT2-deficient HCC cells and their matched control HCC cells were used in the in vivo tumorigenesis assay. **(A)** IHC showed that, compared with the control group, the ANGPT2-overexpressing group had a high ANGPT2 level in tumor tissues, and the ANGPT2-deficient group had a low ANGPT2 level in the tumor tissues. **(B)** Immunoblotting showed that, compared with the control group, the ANGPT2-overexpressing group had a high ANGPT2 level in serum-exosomes, and the ANGPT2-deficient group had a low ANGPT2 level in serum-exosomes.

**Additional file 7: Figure S4.** HCC cell-secreted exosomes promote the angiogenesis capability of HUVECs in vitro. **(A, B)** HUVECs were cultured with or without exosomes derived from Hep3B or MHCC97H cells for 12 h. The Matrigel microtubule formation assay (A) and transwell migration assay(B) showed that HCC cell-secreted exosomes significantly promoted the tubule formation and migration of HUVECs, and MHCC97H-exosomes had a more obvious effect than Hep3B-exosomes. **(C)** HUVECs were cultured with or without HCC cell-secreted exosomes for 48 h, and the wound area was measured at 0, 24 and 48 h. The wound healing assay showed that HCC cell-secreted exosomes led to a significant increase in HUVEC migration, and the effect of MHCC97H-exosomes was more obvious than that of Hep3B-exosomes. **(D)** HUVECs were cultured with or without HCC cell-secreted exosomes for 7 d and were counted by measuring the OD at 450 nm at 1, 3, 5, and 7 d. CCK-8 showed that HUVEC proliferation was significantly increased after coculture with HCC cell-secreted exosomes, and the effect of MHCC97H-exosomes was more significant than that of Hep3B-exosomes. Scale bar = 200 μm (A). *n* = 6 for each group (A, B), *n* = 4 for each group (C, D), *P < 0.05, **P < 0.01, ***P < 0.001, one-way ANOVA with Tukey’s multiple comparison tests.

**Additional file 8: Figure S5.** HCC cell-secreted exosomal ANGPT2 promotes the migration of HUVECs in vitro. HUVECs were cultured with or without HCC cell-secreted exosomes for 48 h, and the wound area was measured at 0, 24 and 48 h. The wound healing assay showed that ANGPT2-overexpressing exosomes led to a significant increase in HUVEC migration, and compared with control exosomes, ANGPT2-deficient exosomes abrogated exosome-induced increase of migration. *n* = 4 for each group, ***P < 0.001, one-way ANOVA with Tukey’s multiple comparison tests.

**Additional file 9: Figure S6.** HCC cell-secreted exosomal ANGPT2 has no obvious effect on the phosphorylation of Tie2 and PI3Kp85. In the time-course experiment, HUVECs were cultured with or without exosomes derived from HCC cells for 15 min, 30 min, 1 h, 2 h, 4 h and 6 h respectively. Immunoblotting showed that the phosphorylation of Tie2 and PI3Kp85 had no obvious changes after coculture with ANGPT2-overexpressing exosomes compared with the coculture with control exosomes.

**Additional file 10: Figure S7.** HCC cell-secreted exosomal ANGPT2 activates the AKT/eNOS and AKT/β-catenin pathways in HUVECs. HUVECs were cultured with or without exosomes derived from HCC cells for 6 h. Immunoblotting showed that ANGPT2-overexpressing exosomes increased the phosphorylation levels of AKT (Ser473 and Thr308), eNOS (Ser1177) and β-catenin in HUVECs, and the promotional effect of ANGPT2-deficient exosomes on the above phosphorylation levels was significantly reduced compared to that of control exosomes. *n* = 4 for each group, *P < 0.05, **P < 0.01, ***P < 0.001, one-way ANOVA with Tukey’s multiple comparison tests.

**Additional file 11: Figure S8.** ANGPT2 promotes migration and proliferation of HCC in vitro. **(A)** The transwell migration assay showed that overexpression of ANGPT2 notably increased the migration of HCC cells, and knockdown of ANGPT2 dramatically decreased HCC cell migration. **(B)** The wound healing assay showed that the migration of ANGPT2-overexpressing HCC cells significantly increased, and the migration of ANGPT2-knockdown HCC cells significantly decreased. **(C)** CCK-8 showed that overexpression of ANGPT2 led to a notable increase in proliferation, and knockdown of ANGPT2 resulted in a significant decrease in HCC cell proliferation. Scale bar = 200 μm. *n* = 5 for each group (A), n = 4 for each group (B, C), ***P < 0.001, Student’s t-tests.

**Additional file 12: Figure S9.** ANGPT2 increases EMT in HCC. **(A, B)** Immunoblotting detected the expression levels of EMT-related proteins. In the in vitro experiment, overexpression of ANGPT2 in HCC cells increased their ZEB1, N-cadherin, vimentin, Twist1 and Snail levels and decreased their E-cadherin levels (A). Knockdown of ANGPT2 decreased the ZEB1, N-cadherin, vimentin, Twist1 and Snail levels and increased the E-cadherin levels (B). n = 4 for each group, **P < 0.01, ***P < 0.001, Student’s t-tests. **(C)** In the in vivo tumorigenesis assay, overexpression of ANGPT2 led to a notable increase in the ZEB1, N-cadherin, vimentin, Twist1 and Snail levels and a significant decrease in the E-cadherin levels, knockdown of ANGPT2 resulted in a dramatic decrease in the ZEB1, N-cadherin, vimentin, Twist1 and Snail levels and an obvious increase in the E-cadherin levels. *n* = 6 for all Hep3B groups, n = 4 for MHCC97H-CT group, n = 5 for other MHCC97H groups, *P < 0.05, **P < 0.01, ***P < 0.001, Student’s t-tests.

**Additional file 13: Figure S10.** ANGPT2 induces angiogenesis via exosomes in HCC. ANGPT2 was delivered into HUVECs from HCC cells via exosome endocytosis and could be recycled by HUVECs. After internalization, HCC cell-secreted exosomal ANGPT2 activated the AKT-eNOS and AKT/β-catenin pathways and induced angiogenesis in HUVECs. Additionally, ANGPT2 increased EMT activation and promoted the malignant progression of HCC.

## Data Availability

The data used or analysed in this study are available from the corresponding author upon reasonable request.

## Abbreviations

ANGPT2: Angiopoietin-2
BLD: Benign liver diseases
CCK-8: Cell counting kit-8 assay
co-IP: Co-immunoprecipitation
DMSO: Dimethylsulfoxide
ELISA: Enzyme-linked immunosorbent assay
EMT: Epithelial-mesenchymal transition
FBS: Fetal bovine serum
HCC: Hepatocellular carcinoma
HUVECs: Human umbilical vein endothelial cells
IHC: Immunohistochemistry
NC: Normal control
NTA: Nanoparticle tracking analysis
PBS: Phosphate buffered saline
PI3Kp85: p85 Subunit of phosphatidylinositol 3-kinase
TEM: Transmission electron microscopy
TME: Tumor microenvironment
