# Supplementary material for: Angiopoietin-2 induces angiogenesis via exosomes in human hepatocellular carcinoma
Source: Cell Commun Signal. 2020 Mar 17;18:46. doi: 10.1186/s12964-020-00535-8 (PMC7077328; doi:10.1186/s12964-020-00535-8)
Supplement: Supplementary file 4 — Additional file 1. Supporting materials and methods. [file 12964_2020_535_MOESM1_ESM.docx]

**Supporting materials and methods**

**Vectors and inhibitors**

The lentivirus pLV-hANGPT2-mCherry, which expressed the ANGPT2-mCherry fusion protein and matched control lentivirus pLV-mCherry, which only expressed the mCherry protein, was generated based on the pLV[Exp]-Puro-EF1A vector (VectorBuilder, USA). For the lentivirus lentiCRISPRv2-ANGPT2gRNA, the sgRNAs were designed using the Zhang laboratory website (http://crispr.mit.edu/): 1. 5’-TCCGCGTTTGCTCCGCTGTT-3’, 2. 5’-TTTCTATCATCACAGCCGTC-3’. Oligos corresponding to the sgRNAs were synthesized and cloned into lentiCRISPRv2 vectors (Addgene, plasmid #52961). lentiCRISPRv2-ANGPT2gRNA constructs, psPAX2 and pMD2.G were cotransfected into HEK293T cells to generate lentiviruses. In addition, lentiCRISPRv2, psPAX2 and pMD2.G were cotransfected into HEK293T cells to generate the control lentivirus lentiCRISPRv2. Lentiviruses were then used to infect Hep3B and MHCC97H cells to obtain stable cell lines by puromycin selection. ANGPT2 overexpression efficiency was detected by qPCR (primer: forward: 5’-CAACACTCAGTGGCTAATGAAG-3’, reverse: 5’-GCATTCTGCTGTATCTCTACCA-3’) and immunoblotting;, ANGPT2 deficient efficiency was detected by genomic DNA sequencing (primer: forward: 5’-AAGATAGGGAGGAGACGACAAACAT-3’, reverse: 5’-CTTTGATTTCTTTCGTGGTGGTGTC-3’) and immunoblotting.

Plasmids pLV-EGFP-hCD63, pLV-EGFP-hRab5 (Genecreate, China) and pLV-EGFP-hRab11 (VectorBuilder, USA), which expressed the CD63-EGFP, Rab5-EGFP and Rab11-EGFP fusion proteins, respectively, were transfected into cells by using Lipofectamine LTX with Plus Reagent (Invitrogen, USA) according to the manufacturer's instructions. Inhibitors nystatin and amiloride (MCE, USA) were dissolved in dimethyl sulfoxide (DMSO) and added to the cells for 30 min before coculture with the exosomes. DMSO was added to the cells as a control. Nystatin, amiloride and control DMSO were also added to culture medium when cells were cocultured with exosomes.

**Exosome isolation and characterization**

Cells were cultured in medium supplemented with 10% exosome-depleted FBS for 48-72 h. Culture medium was collected and centrifuged at 300 × g for 10 min and then 2,000 × g for 20 min. Next, the supernatant was filtered through a 0.22 μm membrane filter and mixed with ExoQuick-TC (EXOTC50A-1, System Biosciences, USA) and then placed at 4°C for 12 h. Finally, the mixture was centrifuged at 1,500 × g for 30 min to isolate the exosome pellet. The serum was filtered through a 0.22 μm membrane filter and mixed with ExoQuick (EXOQ5A-1, System Biosciences, USA) and then placed at 4°C for 30 min. Next, the mixture was centrifuged at 1,500 × g for 30 min to isolate the exosome pellet. Isolated exosome pellets from 10 mL culture medium were resuspended in 1 mL PBS for measuring size by NTA (Nanosight NS300, Malvern, UK), resuspended in 10 μL PBS for TEM (JEM-1400, JEOL, Japan), or resuspended in 5 mL medium for coculture with cells. Isolated exosome pellets from 10 mL culture medium or 100 μL serum were lysed in 200 μL lysis buffer for immunoblotting analysis. Isolated exosome pellets from 100 μL serum were lysed in 100 μL nondenatured protein solubilization reagent for ELISA.

**Immunoblotting and co-IP**

For immunoblotting, proteins were extracted from exosome pellets, cells and tissues using radio immunoprecipitation assay (RIPA) lysis buffer (Beyotime, China) containing a protease-inhibitor and phosphatase-inhibitor (Sigma, USA). Then, the protein concentration was determined by a BCA protein assay kit (Thermo Fisher Scientific, USA), and the content was separated into equal amounts (30 μg for cells, 60 μg for exosomes and tissues), fractionated by sodium dodecyl sulfate polyacrylamide gel electrophoresis, electrotransferred onto a polyvinylidene fluoride membrane (Millipore, USA), and blocked with 1 × tris-buffered saline-tween (TBST) containing 5% nonfat dried milk at room temperature for 1 h. The membranes were incubated at 4°C overnight with the following primary antibodies: anti-Alix (1:200, sc-99010, Santa Cruz, USA), anti-HSP90 (1:200, sc-13119, Santa Cruz, USA), anti-TSG101 (1:500, A1692, ABclonal, China), anti-CD63 (1:200, sc-51662, Santa Cruz, USA), anti-ANGPT2 (1:500, A0698, ABclonal, China), anti-Tubulin (1:2,000, #2146, Cell Signaling Technology, USA), anti-ZEB1(1:1,000, #3396, Cell Signaling Technology, USA), anti-E-cadherin (1:1,000, #14472, Cell Signaling Technology, USA), anti-N-cadherin (1:1,000, #13116, Cell Signaling Technology, USA), anti-Vimentin (1:1,000, #5741, Cell Signaling Technology, USA), anti-Twist1 (1:1,000, #46702, Cell Signaling Technology, USA), anti-Snail (1:1,000, #3879, Cell Signaling Technology, USA), anti-mCherry (1:1,000, YM3125, Immunoway, USA), anti-phospho-Tie2(1:1,000, #4221, Cell Signaling Technology, USA), anti-Tie2 (1:1,000, #4224, Cell Signaling Technology, USA), anti-phospho-PI3Kp85 (1:500, AP0854, ABclonal, China), anti-PI3Kp85 (1:500, A11526, ABclonal, China), anti-phospho-eNOS (Ser1177) (1:500, #9570, Cell Signaling Technology, USA), anti-eNOS (1:1,000, #5880, Cell Signaling Technology, USA), anti-phospho-β-catenin (1:500, #9561, Cell Signaling Technology, USA), anti-β-catenin (1:1,000, #8480, Cell Signaling Technology, USA), anti-phospho-AKT (Ser473) (1:1,000, #4060, Cell Signaling Technology, USA), anti-phospho-AKT (Thr308) (1:1,000, #13038, Cell Signaling Technology, USA), anti-AKT (pan) (1:2,000, #2920, Cell Signaling Technology, USA), anti-GAPDH (1:2,000, #2118, Cell Signaling Technology, USA), anti-CD31 (1:500, YT6076, Immunoway, USA), anti-CD105 (1:500, YM6611, Immunoway, USA), and anti-VEGFA (1:500, A0280, ABclonal, China). Then, the membranes were incubated with the corresponding secondary antibodies (anti-rabbit, 1:2,000, #7074, Cell Signaling Technology, USA; anti-mouse, 1:2,000, #7076, Cell Signaling Technology, USA) for 1 h at room temperature. The quantification was performed using ImageJ (Wayne Rasband National Institutes of Health, USA).

For co-IP, cells were lysed with IP lysis buffer (Beyotime, China) containing a protease-inhibitor and phosphatase-inhibitor (Sigma, USA). The protein was separated out 20 μg as control input. The rest proteins was separated into 200 μg per 200 μL lysates, added the primary antibody anti-mCherry (1:100, YM3125, Immunoway, USA) or control anti-IgG (1:200, AC011, ABclonal, China) and inculated at 4°C overnight, then added 20 μL protein A/G agarose beads (Pierce, USA) and incubated at 4°C for 4 h. The agarose beads were washed with IP washing buffer for 3 times, then added 20 μl of 2 × loading buffer for denaturation of immunoprecipitated proteins. The primary antibodies anti-mCherry (1:1,000, YM3125, Immunoway, USA), anti-Rab5 (1:500, A1180, ABclonal, China), and anti-Rab11 (1:1,000, #5589, Cell Signaling Technology, USA) were used to detected the interaction of ANGPT2-mCherry fused protein with Rab5 or Rab11 by immunoblotting.

**TEM**

Isolated exosome pellets from 10 mL culture medium were resuspended in 10 μL PBS and fixed with 10 μL paraformaldehyde (4%), absorbed onto the formvar-carbon-coated copper grids for 10 min and dried by filter paper; pellets were then stained with 10 μL uranyl acetate (2%) for 90 s and dried, finally examined using TEM (JEM-1400, JEOL, Japan) operated at 120 kV. For immunogold labeling, fixed exosomes were absorbed onto the formvar-carbon-coated copper grids for 30 min, washed with PBS and 50 mM glycine three times respectively, then blocked with 5% bovine serum albumin for 30 min and incubated with the primary antibody (anti-CD63, 1:10, sc-51662, Santa Cruz, USA; anti-ANGPT2, 1:20, SAB3500709, Sigma, USA) for 1 h, washed with 0.5% bovine serum albumin six times and incubated with IgG-gold antibody (anti-mouse 5 nm, 1:20, G7527, Sigma, USA; anti-rabbit 10 nm, 1:20, G7402, Sigma, USA) for 30 min, washed with 0.5% bovine serum albumin and PBS six times respectively, placed on the 100 μL glutaraldehyde (1%) for 2 min and washed with distilled water six times, then stained with 10 μL uranyl acetate (2%) for 90 s and dried, finally examined using TEM. All of the solutions mentioned above were filtered through a 0.22 μm membrane filter, and each wash above was 100 μL solutions and 3 min.

**IHC**

For the clinical study, a total of 96 tissue samples were obtained from HCC patients, and 11 were obtained from BLD patients who underwent hepatectomy at Sun Yat-sen Memorial Hospital at Sun Yat-sen University (Guangzhou, China) between December 2009 and September 2012. For the in vivo tumorigenicity assay, a total of 24 tissue samples were obtained from Hep3B xenografts in nude mice, and 19 were obtained from MHCC97H xenografts in nude mice. IHC assays were performed as previously described.[1] The antibodies used were as follows: anti-ANGPT2 (1:100, A0698, ABclonal, China), anti-CD31 (1:200, GB12063, Servicebio, China). The quantification was performed using Image-Pro Plus 6.0 (Media Cybernetics, USA).

**ELISA**

A total of 67 serum samples were collected from HCC patients, and 26 were collected from BLD patients at Sun Yat-sen Memorial Hospital, Sun Yat-sen University (Guangzhou, China), between 2016 and June 2019. Isolated exosome pellets from 100 μL serum were lysed in 100 μL nondenatured protein solubilization reagent (Invent, China) containing a protease-inhibitor and phosphatase-inhibitor (Sigma, USA), and then the ANGPT2 levels were determined using an ELISA kit according to the manufacturer's instructions (RK00227, ABclonal, China).

**Immunofluorescence and confocal laser scanning microscopy**

HUVECs cultured with exosomes were washed with PBS and fixed with 4% PFA for 15 min, permeabilized with 0.3% Triton X-100 for 15 min and blocked with 10% goat serum for 1 h at room temperature. HUVECs were then incubated with the primary antibody (anti-Rab11, 1:100, #5589, Cell Signaling Technology, USA) for 12 h at 4°C, then incubated with the Alexa Fluor-labeled secondary antibody (1:200, #4416, Cell Signaling Technology, USA) for 1 h at room temperature. HUVECs were incubated with DAPI (Sigma, USA) for 5 min to stain the nuclei and were finally imaged with confocal laser scanning microscope (LV3000, Olympus, Japan). HUVECs that could be imaged directly were fixed with 4% PFA, permeabilized with 0.3% Triton X-100, incubated with DAPI, and finally imaged with a confocal laser scanning microscope. Living HUVECs were directly imaged with a confocal laser scanning microscope for 5 min to obtain 50 images. Fluorescence quantification was performed using ImageJ.

**Matrigel microtubule formation assay**

Then, 100 μL of Matrigel (BD, USA) was coated in each 48-well plate at 37°C for 1 h. Then, 1 × 10^5^ HUVECs were seeded on Matrigel, cultured with 200 μL of FBS-free medium with or without exosomes for 12 h, and imaged with an optical microscope. The length of the tubular structures was quantified by using ImageJ.

**Transwell migration assay**

A total of 1 × 10^5^ cells cultured in 200 μL FBS-free medium with or without exosomes were placed on the upper chamber of a 24-well transwell chamber (8 μm pore size, BD, USA), while the lower chamber was filled with 400 μL 10% FBS-medium. After 12 h of incubation, the cells on the upper side were scraped with a cotton swab, and the cells on the lower side were fixed with 4% PFA for 15 min, stained with crystal violet for 15 min, washed with PBS and dried, then imaged with an optical microscope (EVOS FL Auto, Life Technologies, USA).

**CCK-8**

Two hundred cells were placed in each 96-well plate, cultured with 100 μL medium with or without exosomes for 7 d, and the cells were counted at 1, 3, 5 and 7 d by a cell counting kit-8 according to the manufacturer's instructions (MCE, USA).

**Wound healing assay**

The 6-well plates overspread by monolayer cells were scraped with a pipette tip, washed with PBS, cultured with FBS-free medium with or without exosomes for 48 h, and imaged at 0, 24 and 48 h with an optical microscope.

**In vivo tumorigenesis assay**

First, 1×10^7^ HCC cells were resuspended in 100 μL PBS and injected subcutaneously into the right flank of 5-week-old male BALB/c nude mice. Vernier calipers were used to measure the size of the tumor once every three days. The tumor volume was calculated as 1/2ab^2^, where a is the long axis and b is the short axis of the tumor. The mice were sacrificed after 26 d.

**References:**

1. Wei JX, Lv LH, Wan YL, Cao Y, Li GL, Lin HM, Zhou R, Shang CZ, Cao J, He H, et al: Vps4A functions as a tumor suppressor by regulating the secretion and uptake of exosomal microRNAs in human hepatoma cells. Hepatology 2015, 61:1284-1294.
