# Supplementary figures and images for: Angiopoietin-2 induces angiogenesis via exosomes in human hepatocellular carcinoma
Source: Cell Commun Signal. 2020 Mar 17;18:46. doi: 10.1186/s12964-020-00535-8 (PMC7077328; doi:10.1186/s12964-020-00535-8)

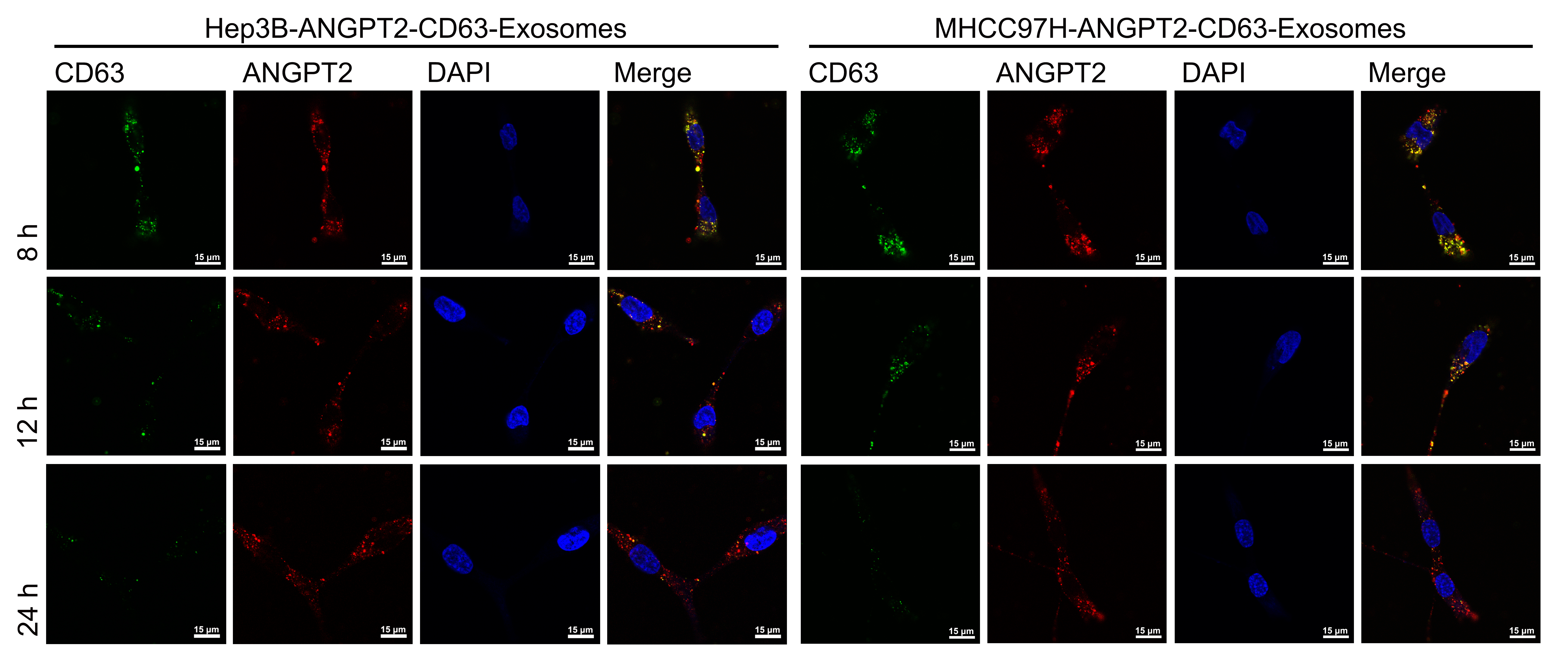

Supplement: Supplementary file 5 — Additional file 2: Figure S1. HCC cell-secreted exosomal ANGPT2 exists in recipient HUVECs for a long time. ANGPT2-mCherry-expressing HCC cells were transfected with pLV-EGFP-CD63 plasmid for 48–72 h to obtain cells that coexpressed the ANGPT2-mCherry and CD63-EGFP fusion proteins. Exosomes isolated from the above HCC cells were cocultured with HUVECs for 6 h and then removed. We observed that ANGPT2-mCherry and CD63-EGFP separated as time went on, and different from exosomal CD63, exosomal ANGPT2 existed in recipient HUVECs up to 24 h by confocal laser scanning microscopy at 8, 12 and 24 h, respectively. Scale bar = 15 μm. [file 12964_2020_535_MOESM2_ESM.jpg]

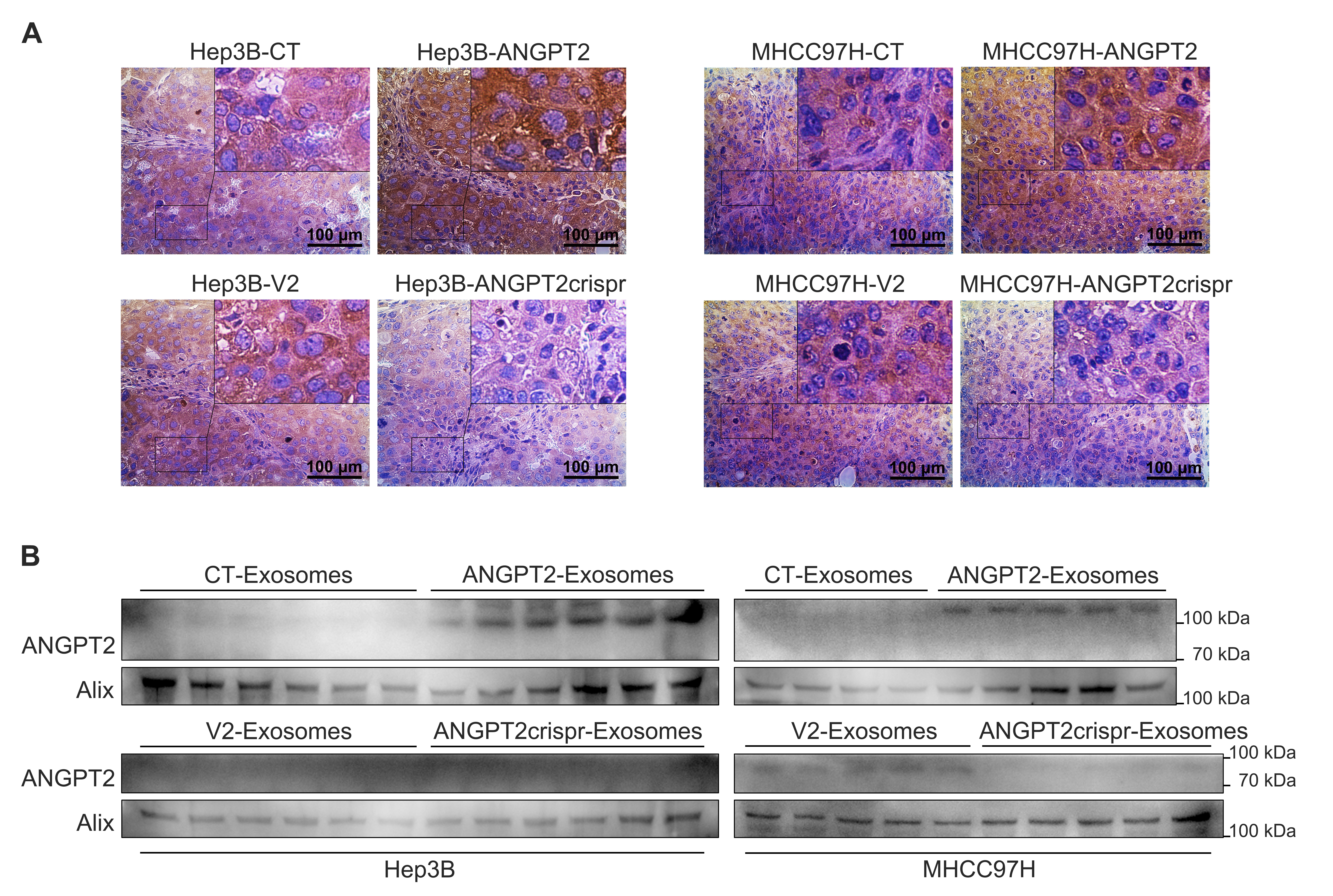

Supplement: Supplementary file 7 — Additional file 6: Figure S3. The overexpression or knockdown of ANGPT2 in HCC tissues and serum-exosomes in vivo. The ANGPT2-overexpressing, ANGPT2-deficient HCC cells and their matched control HCC cells were used in the in vivo tumorigenesis assay. (A) IHC showed that, compared with the control group, the ANGPT2-overexpressing group had a high ANGPT2 level in tumor tissues, and the ANGPT2-deficient group had a low ANGPT2 level in the tumor tissues. (B) Immunoblotting showed that, compared with the control group, the ANGPT2-overexpressing group had a high ANGPT2 level in serum-exosomes, and the ANGPT2-deficient group had a low ANGPT2 level in serum-exosomes. [file 12964_2020_535_MOESM6_ESM.jpg]

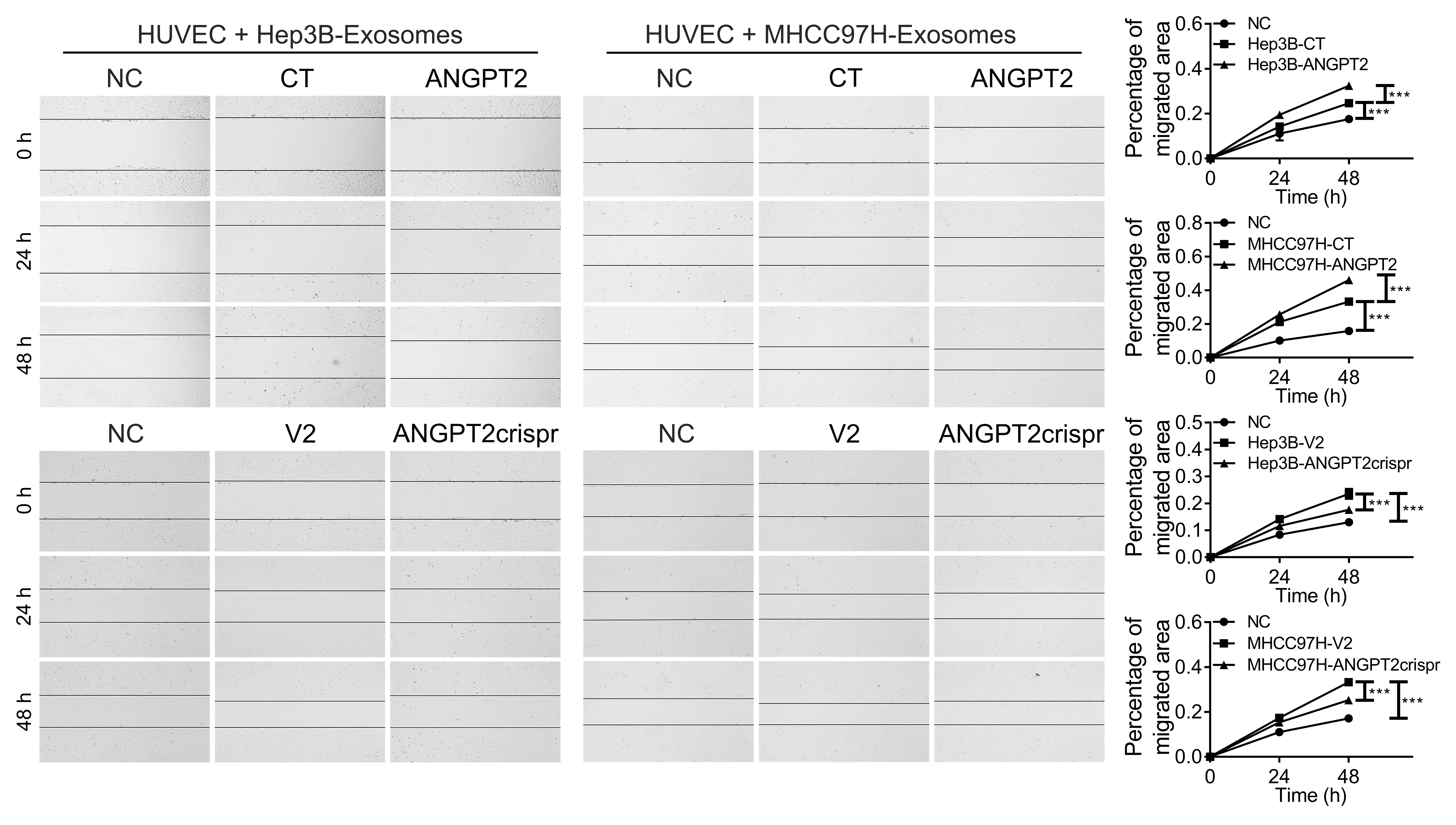

Supplement: Supplementary file 9 — Additional file 8: Figure S5. HCC cell-secreted exosomal ANGPT2 promotes the migration of HUVECs in vitro. HUVECs were cultured with or without HCC cell-secreted exosomes for 48 h, and the wound area was measured at 0, 24 and 48 h. The wound healing assay showed that ANGPT2-overexpressing exosomes led to a significant increase in HUVEC migration, and compared with control exosomes, ANGPT2-deficient exosomes abrogated exosome-induced increase of migration. n = 4 for each group, ***P < 0.001, one-way ANOVA with Tukey’s multiple comparison tests. [file 12964_2020_535_MOESM8_ESM.jpg]

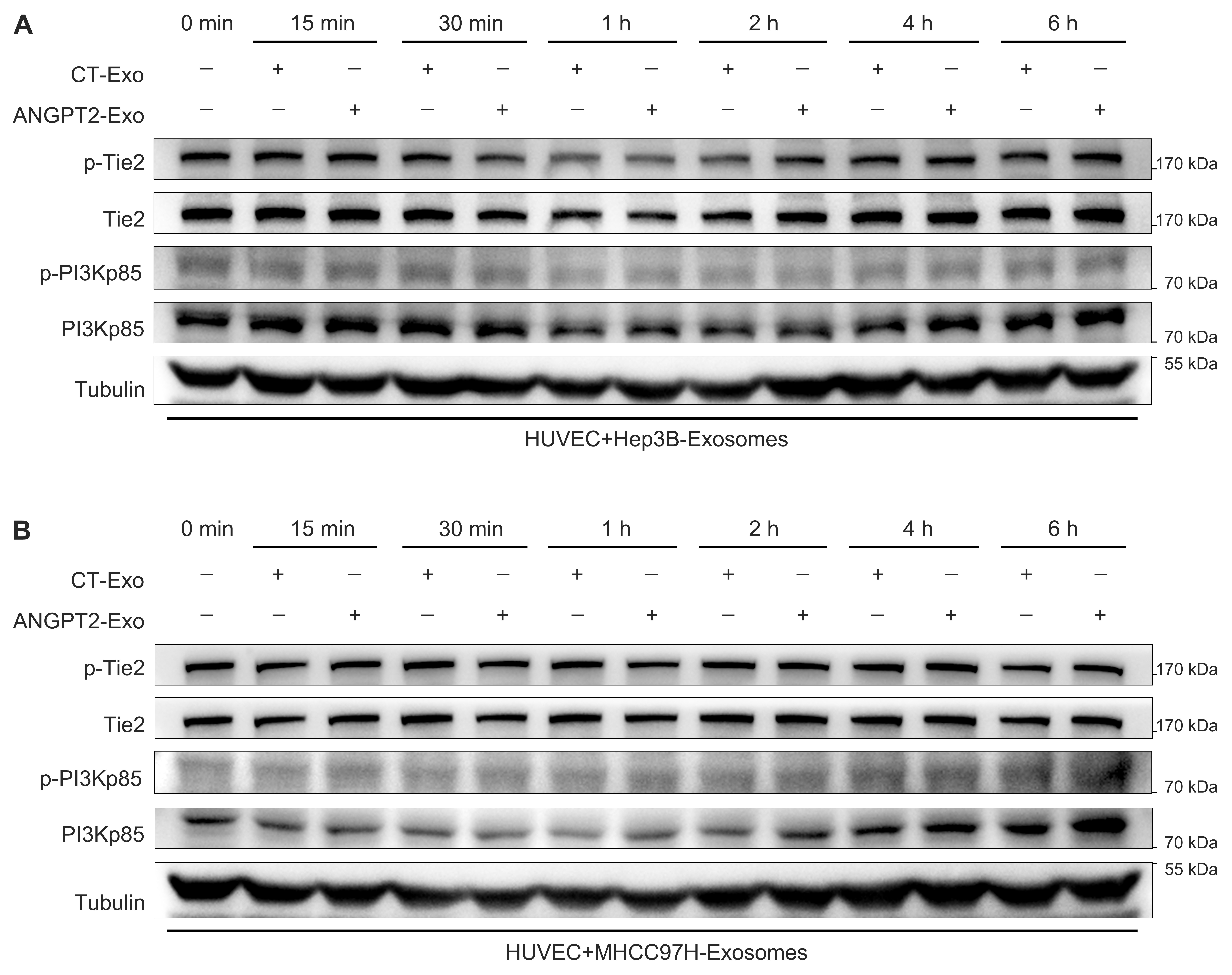

Supplement: Supplementary file 10 — Additional file 9: Figure S6. HCC cell-secreted exosomal ANGPT2 has no obvious effect on the phosphorylation of Tie2 and PI3Kp85. In the time-course experiment, HUVECs were cultured with or without exosomes derived from HCC cells for 15 min, 30 min, 1 h, 2 h, 4 h and 6 h respectively. Immunoblotting showed that the phosphorylation of Tie2 and PI3Kp85 had no obvious changes after coculture with ANGPT2-overexpressing exosomes compared with the coculture with control exosomes. [file 12964_2020_535_MOESM9_ESM.jpg]

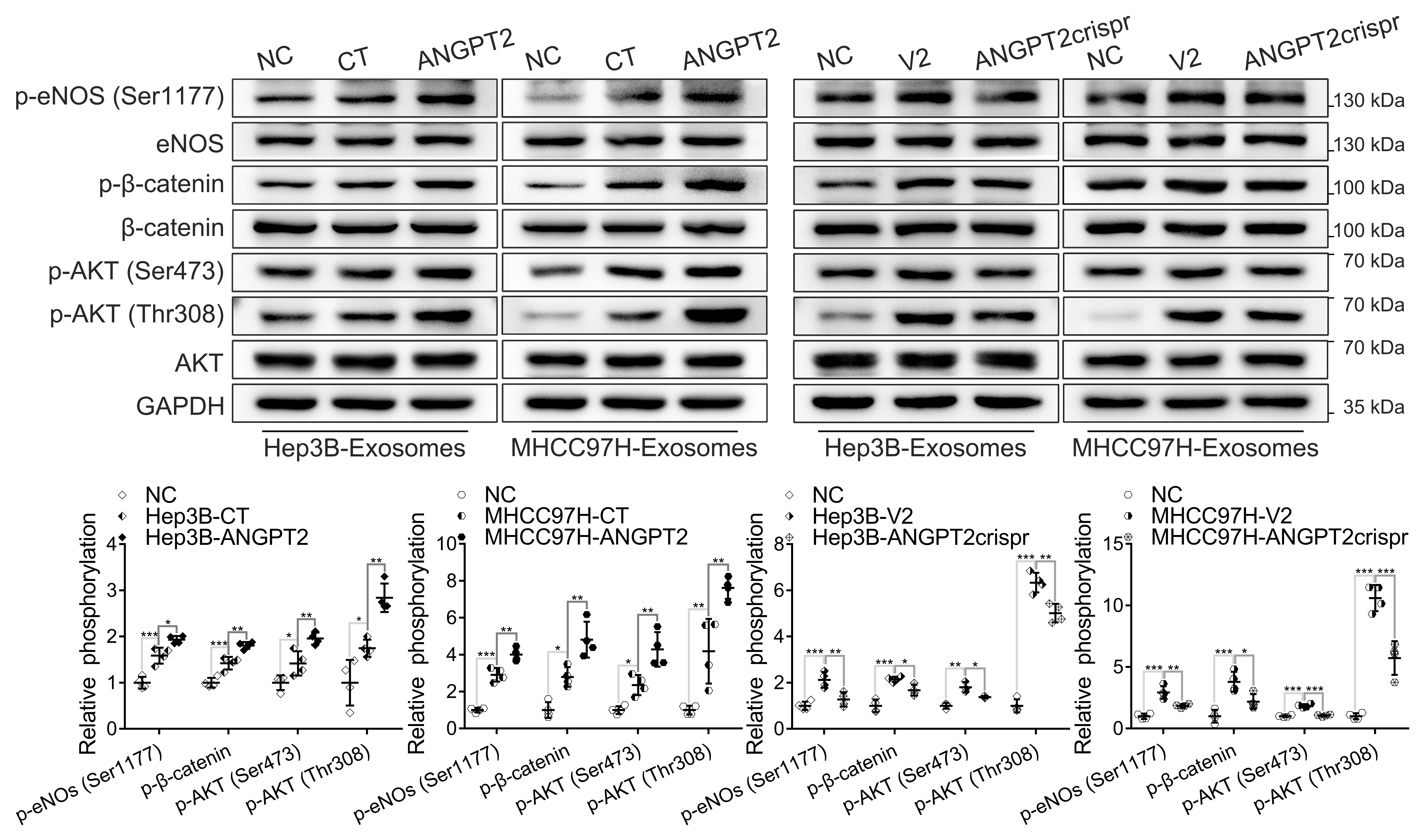

Supplement: Supplementary file 11 — Additional file 10: Figure S7. HCC cell-secreted exosomal ANGPT2 activates the AKT/eNOS and AKT/β-catenin pathways in HUVECs. HUVECs were cultured with or without exosomes derived from HCC cells for 6 h. Immunoblotting showed that ANGPT2-overexpressing exosomes increased the phosphorylation levels of AKT (Ser473 and Thr308), eNOS (Ser1177) and β-catenin in HUVECs, and the promotional effect of ANGPT2-deficient exosomes on the above phosphorylation levels was significantly reduced compared to that of control exosomes. n = 4 for each group, *P < 0.05, **P < 0.01, ***P < 0.001, one-way ANOVA with Tukey’s multiple comparison tests. [file 12964_2020_535_MOESM10_ESM.jpg]

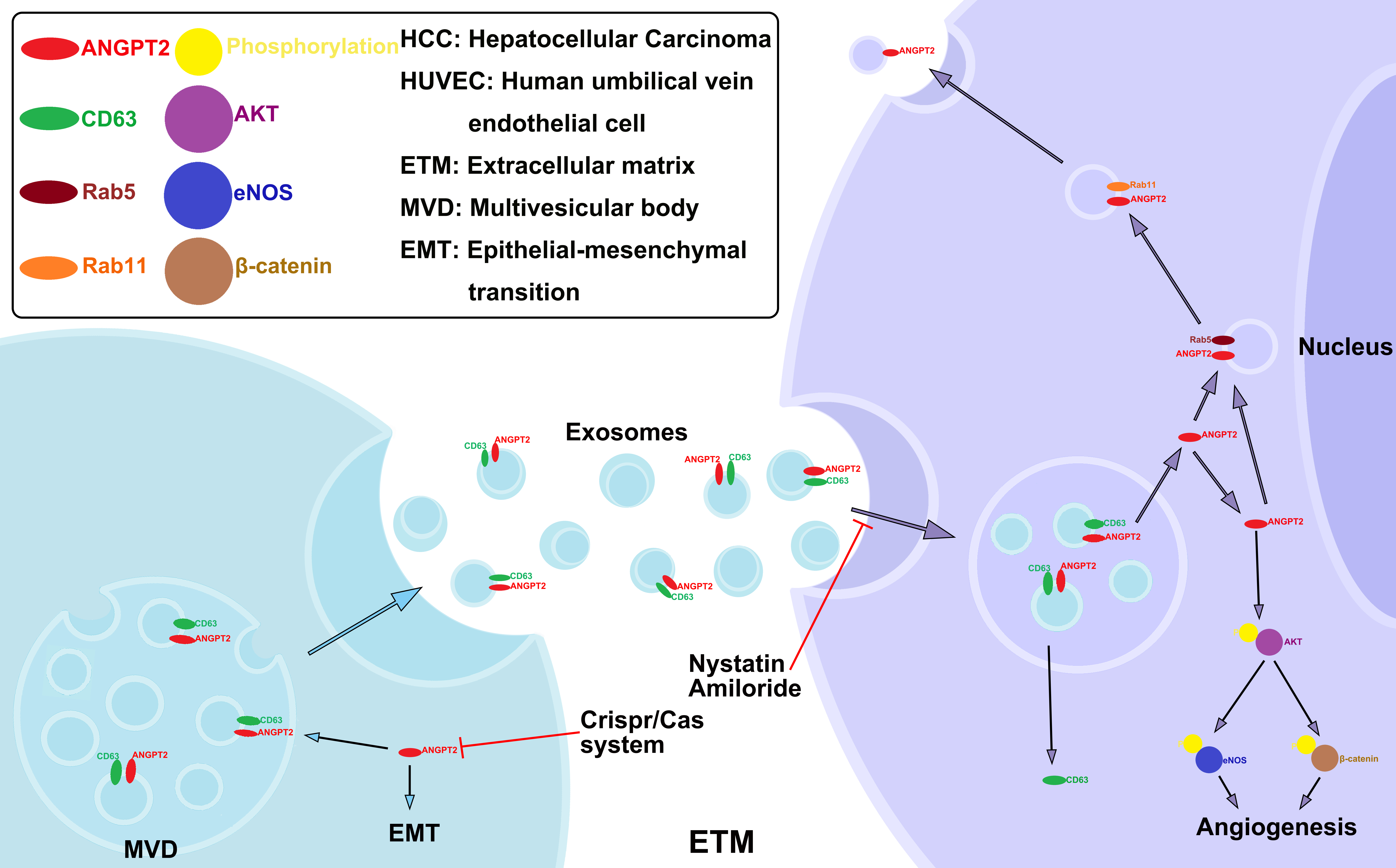

Supplement: Supplementary file 14 — Additional file 13: Figure S10. ANGPT2 induces angiogenesis via exosomes in HCC. ANGPT2 was delivered into HUVECs from HCC cells via exosome endocytosis and could be recycled by HUVECs. After internalization, HCC cell-secreted exosomal ANGPT2 activated the AKT-eNOS and AKT/β-catenin pathways and induced angiogenesis in HUVECs. Additionally, ANGPT2 increased EMT activation and promoted the malignant progression of HCC. [file 12964_2020_535_MOESM13_ESM.jpg]
